# Non-canonical activation of DAPK2 by AMPK constitutes a new pathway linking metabolic stress to autophagy

**DOI:** 10.1038/s41467-018-03907-4

**Published:** 2018-05-01

**Authors:** Ruth Shiloh, Yuval Gilad, Yaara Ber, Miriam Eisenstein, Dina Aweida, Shani Bialik, Shenhav Cohen, Adi Kimchi

**Affiliations:** 1 Department of Molecular Genetics, Weizmann Institute of Science, Rehovot, 76100 Israel; 2 Department of Chemical Research Support, Weizmann Institute of Science, Rehovot, 76100 Israel; 3 Faculty of Biology, Technion Israel Institute of Technology, Haifa, 32000 Israel

## Abstract

Autophagy is an intracellular degradation process essential for adaptation to metabolic stress. DAPK2 is a calmodulin-regulated protein kinase, which has been implicated in autophagy regulation, though the mechanism is unclear. Here, we show that the central metabolic sensor, AMPK, phosphorylates DAPK2 at a critical site in the protein structure, between the catalytic and the calmodulin-binding domains. This phosphorylation activates DAPK2 by functionally mimicking calmodulin binding and mitigating an inhibitory autophosphorylation, providing a novel, alternative mechanism for DAPK2 activation during metabolic stress. In addition, we show that DAPK2 phosphorylates the core autophagic machinery protein, Beclin-1, leading to dissociation of its inhibitor, Bcl-X_L_. Importantly, phosphorylation of DAPK2 by AMPK enhances DAPK2’s ability to phosphorylate Beclin-1, and depletion of DAPK2 reduces autophagy in response to AMPK activation. Our study reveals a unique calmodulin-independent mechanism for DAPK2 activation, critical to its function as a novel downstream effector of AMPK in autophagy.

## Abstract (web summary)

DAPK2 is a calmodulin-regulated protein kinase implicated in autophagy regulation, but how physiological stress leads to its activation is yet unknown. Here, the authors show that the central metabolic sensor AMPK phosphorylates DAPK2 to promote autophagy in a calmodulin-independent mechanism.

## Introduction

Autophagy is an evolutionarily conserved, highly regulated intracellular bulk degradation process in which a double-membrane vesicle, called an autophagosome, is formed in the cytosol and encloses cytosolic material. The autophagosome later fuses with the lysosome, where its content is degraded (reviewed in refs. ^1,2^). Autophagy is involved in many cellular events, including development, immune defense, programmed cell death, and neuronal degeneration^3^, and is a physiological response to fasting. Under steady-state conditions, basal levels of autophagy are maintained within the cell. Under certain stress conditions, such as nutrient deprivation, autophagy is stimulated in order to ensure cell survival by providing the components needed to maintain homeostasis^4^. The first step in autophagosome biogenesis, termed the nucleation step, is mediated by a class III PI(3)K multi-protein complex containing Beclin-1, Atg14, Vps15, and the lipid kinase Vps34^5,6^. This complex is responsible for the production of PI(3)P at the autophagosome assembly site, which in turn serves to recruit other autophagic proteins. The autophagy-promoting activity of Beclin-1 is suppressed, among other mechanisms, by direct binding of anti-apoptotic members of the Bcl-2 family to the BH3 domain of Beclin-1^7^.

Death-associated protein kinase 2 (DAPK2; also named DRP-1) is a 42-kDa Ca^2+^/calmodulin (CaM)-regulated Ser/Thr kinase^8^ and a member of the DAPK family (for a review see ref. ^9^). The founder member of the family is DAPK1, a large multi-domain Ca^2+^/CaM-regulated Ser/Thr kinase implicated in a wide range of biological activities (for a review see ref. ^10^). Both DAPK1 and DAPK2 show a high degree of homology in their N-terminal catalytic domains and the adjacent CaM auto-regulatory domains. Yet they differ substantially in the structure of their extra-catalytic domains, resulting in different intracellular localizations and different profiles of interacting proteins^9,10^. In DAPK2, a short C-terminal tail of 40 amino acids follows the kinase and the CaM auto-regulatory domains. The crystal structure of DAPK2 appears as a homodimer in an auto-inhibited conformation, blocking substrate access^11^. Homodimerization is mediated by a basic loop in the catalytic domain^11^, which is a signature of the DAPK family, and by the C-terminal tail^12^.

DAPK2 mediates a range of cellular processes (for a recent review see ref. ^13^), including myeloid differentiation^14^, erythroblast loss during erythropoiesis^15^, granulocyte motility^16^, and anoikis^17^. Another prominent function of DAPK2, which it shares with DAPK1, is its link to autophagy, initially discovered in overexpression experiments, in which DAPK2 triggered the appearance of double-membrane autophagic vesicles^18^. Subsequent knock-down experiments confirmed the involvement of DAPK2 in regulating autophagy in human cell lines^19^ and preadipocytes^20^.

A screen for interacting proteins within the cell death network identified Atg14 and several isoforms of 14-3-3 as interactors of DAPK2^21^. A subsequent proteomics screen identified tubulin^22^. More recently, a proteomics screen by mass spectrometry, followed by validation with bimolecular fluorescence complementary assays, identified α-actinin and 14-3-3-β as DAPK2 interacting proteins^23^.

Despite the numerous functions attributed to DAPK2, only a limited number of DAPK2 substrates has been identified. Its first known substrate was myosin II regulatory light chain. Recently, DAPK2 was found to phosphorylate the mTOR binding partner raptor^19^, and the autophagy receptor protein p62/SQSTM^24^.

Consistent with its various functional roles, it is not surprising that DAPK2 activity is tightly regulated in cells. In the absence of CaM, the auto-regulatory domain binds to the catalytic cleft, blocking access of exogenous substrates and inhibiting DAPK2 function. Autophosphorylation of Ser308 within the CaM auto-regulatory domain reinforces this inhibitory mechanism by stabilizing the docking of the CaM auto-regulatory domain in the catalytic cleft^12,25^. Thus, the canonical mechanism for DAPK2 activation requires binding of CaM, which pulls the CaM auto-regulatory domain out of the catalytic cleft, and dephosphorylation of Ser308. An additional regulatory mechanism is phosphorylation of one or more of the last 4 amino acids of the protein (SSTS), which constitutes a 14-3-3 binding site. Phosphorylation and consequent 14-3-3 binding inhibit DAPK2 activity^21^. The kinase(s) responsible for this inhibitory phosphorylation are yet unknown, though AKT was proposed as a possible candidate^26^.

In this study, we identify the metabolic sensor AMP-activated protein kinase (AMPK) as an upstream activator of DAPK2. AMPK is a key regulator of energy homeostasis in eukaryotic cells^27^. Once activated, it acts to suppress anabolic pathways and promote catabolic pathways, such as autophagy. We show that AMPK phosphorylates DAPK2 on Ser289 in cell cultures exposed to metabolic stress and in muscle tissue of fasted mice. Most interestingly, we demonstrate that this single phosphorylation of Ser289, localized at a critical site in the DAPK2 structure, provides a novel non-canonical mechanism of DAPK2 activation that is independent of CaM binding. It mitigates the Ser308 auto-inhibitory phosphorylation and reduces DAPK2 homodimerization, two activating events that most likely result from a conformational change imposed by Ser289 phosphorylation. In addition, we show that DAPK2 phosphorylates the core autophagic machinery protein, Beclin-1, on Thr119 located in its BH3 domain, thus causing it to dissociate from its inhibitor Bcl-X_L_. The importance of this novel AMPK–DAPK2 axis in autophagy regulation is further substantiated by the findings that phosphorylation of DAPK2 by AMPK enhances DAPK2’s ability to phosphorylate Beclin-1, and that depletion of DAPK2 reduces autophagy in response to AMPK activation. Altogether, these results suggest that DAPK2 is an important downstream effector of AMPK in promoting autophagy.

## Results

### AMPK phosphorylates DAPK2 on Ser289

To search for possible kinases that regulate DAPK2, the amino acid sequence of DAPK2 was analyzed using the Scansite algorithm^28^ at high stringency, looking for all potential motifs. Four potential phosphorylation sites were found (Tyr208, Thr265, Ser289, Thr359). Next, this list was crossed with the PhosphoSitePlus database^29^ for reported phosphorylation sites on DAPK2. Two sites were found in both lists: Ser289 and Thr359. According to the Scansite algorithm, both Ser289 and Thr359 fit the consensus sequence of AMPK, and Ser289 also fits the consensus sequence of PKA. We focused on AMPK as a possible upstream regulator of DAPK2, as both AMPK and DAPK2 are known to promote autophagy. Therefore, we hypothesized that they may act together through a signaling cascade.

**Fig. 1:**
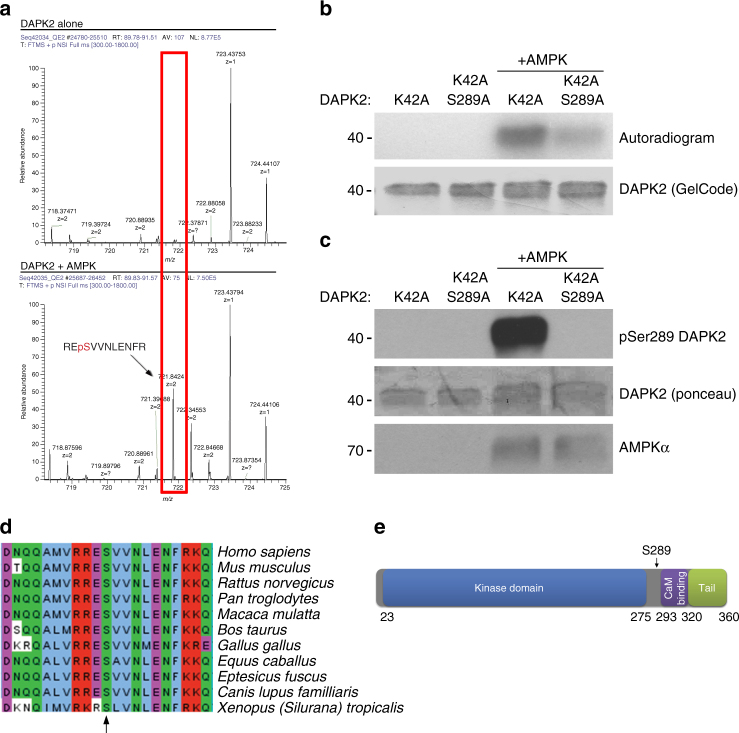
AMPK phosphorylates DAPK2 on Ser289. **a** DAPK2-K42A was subjected to an in vitro kinase assay with AMPK and resolved by SDS–PAGE. A sample without AMPK was used as control. The gel was stained with GelCode Blue and the DAPK2 bands in each sample were excised and analyzed by liquid chromatography-mass spectrometry/mass spectrometry (LC–MS/MS) as shown in the graphs. Each peak represents a peptide; the graph height represents the relevant abundance of the peptide in the fraction. **b** AMPK was incubated with either DAPK2 K42A or DAPK2 K42A S289A in a kinase reaction mixture containing ^32^P-labeled ATP. Reactions were resolved by SDS–PAGE and exposed to X-ray film. **c** AMPK was incubated with either DAPK2 K42A or DAPK2 K42A S289A in a kinase reaction mixture. Reactions were resolved by SDS-PAGE and reacted with an antibody raised against pSer289 DAPK2. **d** Multiple sequence alignment of different DAPK2 orthologues using ClustalOmega. Ser289 is marked with an arrow. **e** A scheme showing the different domains of DAPK2. Ser289 is marked with an arrow

To test if AMPK can directly phosphorylate DAPK2, we performed an in vitro kinase assay using recombinant proteins and analyzed it by mass spectrometry for phospho-peptide mapping. The analysis revealed that DAPK2 was indeed phosphorylated by AMPK, and that the only site that was phosphorylated at high confidence was Ser289 (Fig. 1a). To further verify this result, a radioactive kinase assay was performed using two DAPK2 mutants as substrates: a K42A catalytically inactive mutant (to abolish autophosphorylation that would mask the trans-phosphorylation signal), and a K42A S289A double-mutant. The DAPK2 K42A mutant was strongly phosphorylated by AMPK, while the K42A S289A double mutant showed a very weak signal (Fig. 1b), further confirming that the major phosphorylation site is Ser289.

Next, an antibody that specifically recognizes DAPK2 when it is phosphorylated on Ser289 was generated. In vitro kinase assays confirmed the specificity of this anti-phospho antibody, which gave a strong signal only when DAPK2 K42A, but not the K42A S289A double mutant, was incubated with AMPK (Fig. 1c). Notably, upon sequence alignment of DAPK2 orthologues (DAPK2 is found only in vertebrates^30^), Ser289, as well as the adjacent residues that constitute the consensus sequence recognized by AMPK, were found to be highly conserved (Fig. 1d), indicating the potential significance of this consensus as a regulatory phosphorylation site. This site resides in a short loop (residues 289–292), critically located between the kinase domain and the CaM-binding domain of DAPK2 (Fig. 1e). As this loop was proposed to act as a flexible hinge that allows a swing-out motion of the CaM binding helix in DAPK1^31^, it became of interest to study the potential regulatory role of phosphorylation at this site.

### DAPK2 is phosphorylated on Ser289 upon AMPK activation

**Fig. 2:**
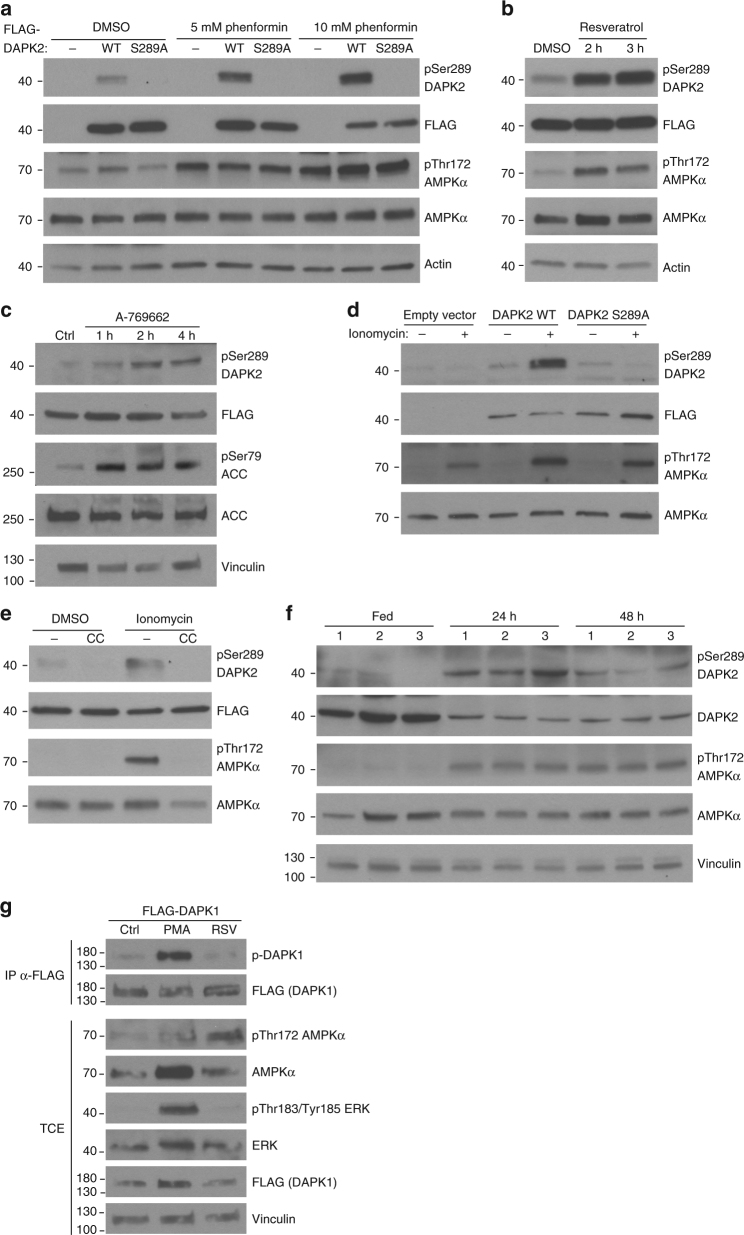
DAPK2 is phosphorylated on Ser289 upon AMPK activation. **a** HCT116 cells were transfected with FLAG-DAPK2 WT, S289A or empty vector and treated with 5 mM/10 mM phenformin or DMSO for 4 h. **b** HCT116 cells were transfected with FLAG-DAPK2 WT and treated with 250 μM resveratrol for 2/3 h. **c** HCT116 cells were transfected with FLAG-DAPK2 WT and treated with 100 μM A-769662 for 1/2/4 h. **d** A549 cells were transfected with FLAG-DAPK2 WT, S289A or empty vector and treated with 5 μM ionomycin for 2 h. **e** A549 cells were transfected with FLAG-DAPK2 and treated with 10μM ionomycin or DMSO for 1 h, with or without the addition of 5 μM compound C. **f** Mice were fasted for either 24 or 48 h, or fed normally, as control. Muscle tissue from three different mice at each condition was extracted and protein lysates were subjected to western blots. **g** HCT116 cells were transfected with FLAG-DAPK1 and treated with 200 µM resveratrol for 2 h or serum-starved overnight and then treated with 100 nM PMA for 30 min. Anti-FLAG immunoprecipitates were resolved by SDS–PAGE. DAPK1 Ser289 phosphorylation was monitored using an antibody that recognizes the sequence RXRXXpSer/Thr, which was previously shown to specifically detect phosphorylation of DAPK1 on Ser289^38^. In all panels, experiments were repeated three times and representative immunoblots are shown

To determine if AMPK can phosphorylate DAPK2 on Ser289 in cells, HCT116 cells were treated with two ATP synthesis inhibitors that act as AMPK activators: phenformin and resveratrol. Both reagents increase cellular AMP/ADP relative to ATP, causing displacement of ATP from the γ subunit of AMPK by AMP/ADP, thus promoting phosphorylation and activation the catalytic subunit by its upstream activator LKB1. Indeed, treatment of HCT116 cells with phenformin led to both AMPK activation (as monitored by the level of AMPK Thr172 phosphorylation) and DAPK2 Ser289 phosphorylation (Fig. 2a). The specificity of the pSer289 DAPK2 antibody in cells was verified using a DAPK2 S289A mutant. Similarly, treatment of HCT116 cells with resveratrol led to both AMPK activation and DAPK2 Ser289 phosphorylation (Fig. 2b). Neither phenformin nor resveratrol induced caspase-dependent apoptosis under these conditions, as no cleavage of caspase-9, caspase-3 or PARP-1 was detected (Supplementary Figure 1a). As both phenformin and resveratrol cause reduction in cellular ATP levels and thus may activate additional signaling pathways, HCT116 cells were treated with the more specific AMPK allosteric activator A-769662. Indeed, A-769662 activated AMPK, as monitored by phosphorylation of its substrate ACC, and also induced DAPK2 Ser289 phosphorylation (Fig. 2c), suggesting that AMPK activation is the direct cause for DAPK2 Ser289 phosphorylation in cells.

Next, this result was further validated and extended to other cells and stimuli. In A549 cells, the upstream kinase LKB1 is mutated, and AMPK can instead be activated by an alternative kinase, CAMKK2, in response to increases in intracellular calcium levels^32,33^. Indeed, increasing calcium levels in A549 cells with ionomycin caused both AMPK activation and DAPK2 Ser289 phosphorylation (Fig. 2d). Moreover, treatment of the cells with compound C, a commonly used inhibitor of AMPK, abolished both AMPK activation and DAPK2 Ser289 phosphorylation in response to ionomycin (Fig. 2e). Ionomycin did not induce caspase-dependent apoptosis under these conditions (Supplementary Figure 1b).

Finally, DAPK2 phosphorylation by AMPK was also assessed in vivo in response to metabolic stress. Mice were fasted for 24 or 48 h, or normally fed as control, and muscle atrophy in the fasted mice was measured by reduction in muscle weight and muscle fiber size (Supplementary Figure 2). Protein extracts that were prepared from muscle tissue were then analyzed by western blotting. Consistent with previous reports^34^, AMPK was activated upon fasting. Interestingly, the total level of DAPK2 protein was reduced in fasted mice, as occurs for many proteins upon muscle atrophy^35–37^. Notably, despite the reduction in the amount of total DAPK2 protein, there was a significant elevation in the level of pSer289 DAPK2 (Fig. 2f). Therefore, DAPK2 is phosphorylated on Ser289 in response to AMPK activation in vivo.

Ser289 is also conserved in DAPK1, and although its surrounding sequence is not identical, it still constitutes a potential AMPK consensus site according to the Scansite algorithm (Supplementary Figure 6). Intriguingly, it was previously reported that DAPK1 is phosphorylated on Ser289 by the kinase RSK in response to activation of the MAPK/ERK survival signaling pathway^38^. Thus, it was interesting to test whether DAPK1 is also phosphorylated by AMPK at this site. Using an antibody that recognizes phosphoSer289 DAPK1, we compared the outcome of these two different signaling pathways. To this end, HCT116 cells were transfected with FLAG-DAPK1 and treated either with PMA to activate the MAPK/ERK pathway, resveratrol to activate AMPK, or left untreated as control. Consistent with previous reports, activation of the MAPK/ERK pathway by PMA, as measured by ERK phosphorylation, induced strong phosphorylation of DAPK1 on Ser289. In contrast, activation of AMPK by resveratrol had no effect on DAPK1 Ser289 phosphorylation (Fig. 2g). Thus, Ser289 phosphorylation by AMPK is unique to DAPK2.

### Ser289 phosphorylation enhances DAPK2 catalytic activity

**Fig. 3:**
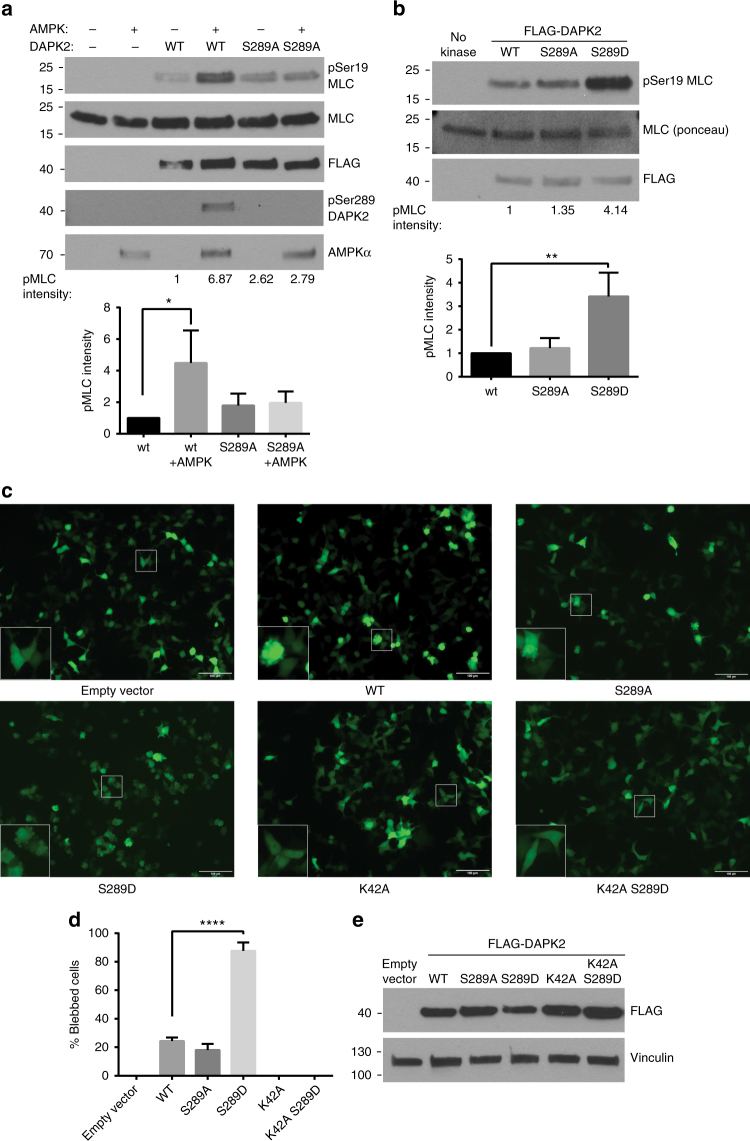
Ser289 phosphorylation enhances DAPK2’s catalytic activity. **a** Sequential kinase assays of AMPK on FLAG-DAPK2 in AMPK kinase buffer, and then of DAPK2 on MLC in DAPK2 kinase assay buffer. Phosphorylation was assessed by western blotting of the reaction mixtures. Bar graph represents pMLC intensity as mean ± SD of three independent repeats. Statistical analyses were performed using one-way ANOVA with post hoc Dunnett’s multiple comparison test. *P < 0.05. **b** FLAG-DAPK2 WT, S289A or S289D was incubated in a kinase reaction mixture with MLC, and phosphorylation assessed by western blotting of the reaction mixtures. pMLC band intensity was quantified using NIH ImageJ software. Bar graph represents pMLC intensity as mean ± SD of three independent repeats. Statistical analyses were performed using one-way ANOVA with post hoc Dunnett’s multiple comparison test. **P < 0.01. **c** HEK293T cells were transfected with GFP and the indicated constructs, and imaged after 24 h. Scale bar=100 µm. **d** Quantification of the extent of blebbing among GFP-positive cells. Bar graph represents percent of blebbed cells as mean ± SD of three independent repeats. Statistical analyses were performed using one-way ANOVA with post hoc Dunnett’s multiple comparison test. ****P < 0.0001. **e** Western blot of a representative experiment from **c**

To test the effect of Ser289 phosphorylation on DAPK2's catalytic activity, FLAG–DAPK2 was incubated with AMPK in a kinase reaction mix containing AMPK kinase assay buffer, thus allowing AMPK to phosphorylate DAPK2. Next, the mix was diluted with DAPK2 kinase assay buffer and recombinant MLC was added as substrate. As a negative control, the same reaction was carried out using the DAPK2 S289A mutant that cannot be phosphorylated by AMPK. MLC phosphorylation was significantly higher when DAPK2 WT was pre-incubated with AMPK and phosphorylated by it on Ser289 (Fig. 3a). In contrast, pre-incubation of DAPK2 S289A with AMPK did not enhance MLC phosphorylation. These results indicate that phosphorylation of DAPK2 on Ser289 enhances its catalytic activity towards MLC.

Next, phospho-silencing and phospho-mimicking mutants were generated for this site (S289A and S289D, respectively). To test if the phospho-mimicking mutation has the same effect as actual phosphorylation by AMPK, FLAG-DAPK2 WT and Ser289 mutants were immunoprecipitated from HEK293T cells and used in an in vitro kinase assay on recombinant MLC. Notably, the Ser289 phospho-mimicking mutant showed significantly higher catalytic activity towards MLC (Fig. 3b), indicating that the S289D mutation indeed mimics the effect of actual phosphorylation in terms of catalytic activity. Therefore, these mutants were further used in cell-based experiments.

Transient transfection of WT DAPK2 in HEK293T cells was previously shown to cause membrane blebbing, most likely as a result of the interaction with α-actinin and phosphorylation of MLC near the plasma membrane^18,23^. Thus the blebbing phenotype can serve as a quantitative measure to assess DAPK2 catalytic activity in cells. HEK293T cells were transfected with either DAPK2 WT or Ser289 mutants, or empty vector as control, together with GFP, and cell morphology was examined by fluorescent microscopy (Fig. 3c). Upon overexpression, the S289D phospho-mimicking mutant induced a significantly higher level of membrane blebbing compared to WT DAPK2 (88% of total GFP-positive cells, compared to 24%) (Fig. 3d). It should be noted that overexpression of the different DAPK2 constructs did not induce cleavage of caspase-3, caspase-9 or PARP1, indicating that blebbing did not result from activation of caspase-dependent apoptosis (Supplementary Figure 3). These results demonstrate that the phospho-mimicking mutation significantly enhances the blebbing phenotype induced by DAPK2. The strong increase in blebbing resulted from enhanced catalytic activity, as the S289D mutation had no effect in these assays when introduced on the background of the K42A inactivating mutation. Equal expression of the different DAPK2 constructs was verified by western blot (Fig. 3e).

### Ser289 phosphorylation leads to CaM-independent activation

**Fig. 4:**
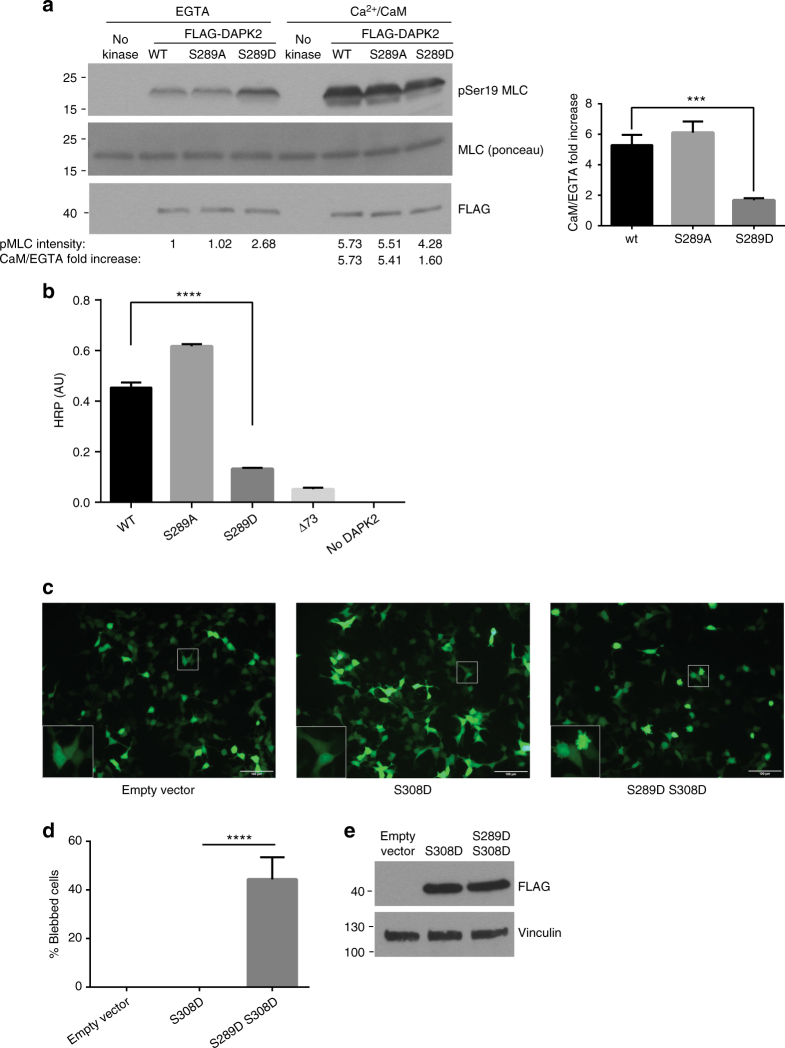
Ser289 phosphorylation provides a CaM-independent mechanism of activation. **a** FLAG-DAPK2 WT, S289A or S289D was incubated in a kinase reaction mixture with MLC. Reactions were carried out with the addition of either EGTA or Ca^2+^/CaM. pMLC band intensity was quantified using NIH ImageJ software. The fold increase ratio upon addition of Ca^2+^/CaM was calculated by dividing the calculated intensity of the Ca^2+^/CaM lanes with the calculated intensity of the respective EGTA lanes. Bar graph represents CaM/EGTA fold increase as mean ± SD of three independent repeats. Statistical analyses were performed using one-way ANOVA with post hoc Dunnett’s multiple comparison test. ***P < 0.001. **b** ELISA plates were coated with CaM and incubated with DAPK2 WT or different mutants. Bar graph represents quantification of DAPK2 binding as mean ± SD of three technical repeats from one representative experiment out of three independent repeats. Statistical analyses were performed using one-way ANOVA with post hoc Dunnett’s multiple comparison test. ****P < 0.0001. **c** HEK293T cells were transfected with GFP and the indicated constructs and imaged 24 h post-transfection. Scale bar=100 µm. **d** Quantification of the extent of blebbing among GFP-positive cells. Bar graph represents percent of blebbed cells as mean ± SD of three biological repeats. Statistical analyses were performed using one-way ANOVA with post hoc Dunnett’s multiple comparison test. ****P < 0.0001. **e** Western blot of a representative experiment from **c**

DAPK2 is a CaM-regulated kinase and is thus activated by calcium signaling in cells. On the other hand, Ser289 phosphorylation is induced by metabolic stress, which is not accompanied by elevation in calcium levels. It was therefore interesting to compare the two mechanisms of activation and test whether they are synergistic or mutually exclusive. To this end, an in vitro kinase assay was performed using the Ser289 mutants under two different conditions: with the addition of Ca^2+^/CaM, or with the calcium chelator EGTA as a control. Interestingly, while the Ser289 phospho-mimicking mutant showed significantly higher catalytic activity towards MLC in the absence of Ca^2+^/CaM (~2.5 fold higher), upon the addition of Ca^2+^/CaM the stimulatory effect of the mutation was lost (Fig. 4a). Thus, the fold induction of the catalytic activity upon addition of Ca^2+^/CaM was significantly lower for the S289D phospho-mimicking mutant than for the WT and the S289A mutant (1.6 versus 5.7 and 5.4, respectively). Therefore, not only is the S289D mutant highly active without the presence of Ca^2+^/CaM, but CaM binding also has a considerably reduced effect on its catalytic activity.

Next, the effect of Ser289 phosphorylation on DAPK2’s affinity to CaM was tested by ELISA. Wells were coated with CaM and then incubated with equal amounts of DAPK2 WT and Ser289 mutants. A DAPK2 mutant lacking the entire CaM auto-regulatory domain (Δ73 mutant) was used as a negative control. Remarkably, the S289D phospho-mimicking mutant gave rise to a greatly reduced signal compared to DAPK2 WT (~5 fold lower) (Fig. 4b), implying that phosphorylation of Ser289 reduces DAPK2’s affinity to CaM. Altogether, these results suggest that Ser289 phosphorylation provides an alternative mechanism for DAPK2 activation that is separate from calcium signaling and CaM binding.

The activating effect of CaM results from pulling the CaM binding domain away from the catalytic cleft and stabilizing the active conformation of the kinase. On the other hand, autophosphorylation of Ser308 secures the docking of the CaM binding domain in the catalytic cleft^12^. The finding that the S289D mutant does not depend on CaM for activation prompted us to test whether Ser289 phosphorylation may functionally mimic the effect of CaM binding and oppose the effect of Ser308 phosphorylation. To this end, HEK293T cells were transfected with an S308D or an S289D S308D double mutant, together with GFP. Upon overexpression, the S308D phosphomimetic mutant was completely silent and did not induce any membrane blebbing (Fig. 4c, d), consistent with previous results^12^. Interestingly, introduction of the S289D mutation mitigated this strong inhibition, as the S289D S308D double mutant induced blebbing in 45% of GFP-positive cells (Fig. 4c, d). Equal expression of the different constructs was verified by western blot (Fig. 4e). This result indicates that the S289D mutation can override the strong inhibitory effect of the S308D mutation on the catalytic activity of DAPK2. Altogether, these results indicate that Ser289 phosphorylation provides a novel mechanism to upregulate DAPK2 activity, independent of CaM binding and Ser308 dephosphorylation.

### S289D mutation reduces dimerization and autophosphorylation

**Fig. 5:**
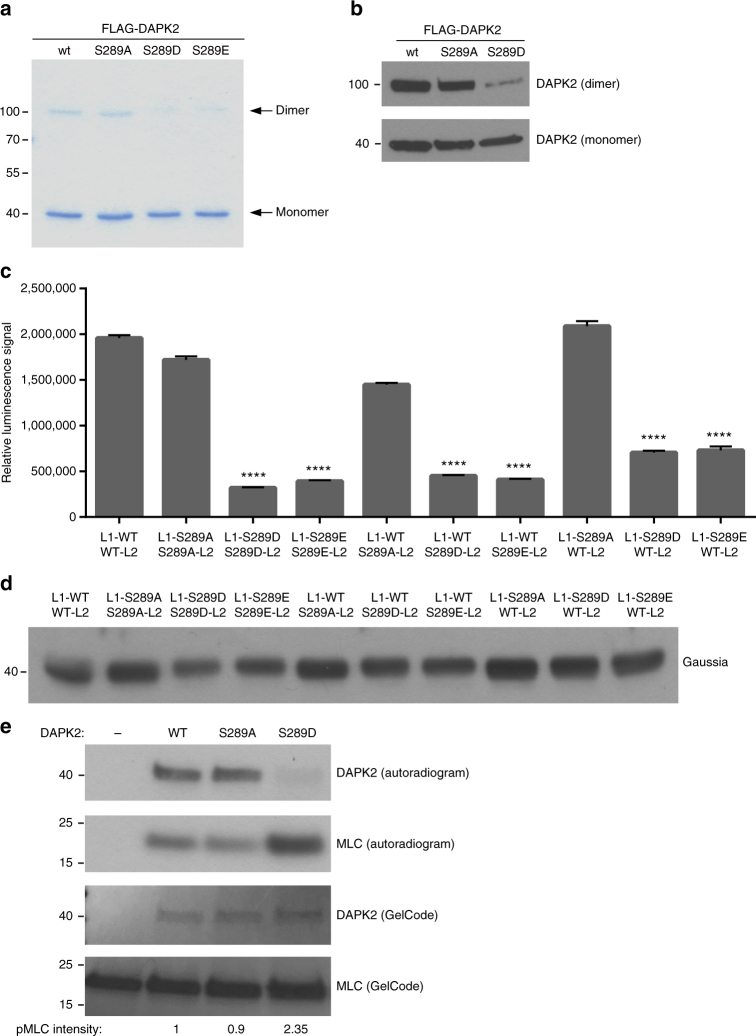
Ser289 phosphorylation reduces dimerization and autophosphorylation. **a** FLAG-DAPK2 WT and mutants were overexpressed in HEK293T cells and immunoprecipitated using anti-FLAG antibody. Immunoprecipitated protein was resolved by SDS-PAGE and the gel was stained with GelCode Blue. A representative gel of three independent experiments is shown. **b** FLAG-DAPK2 WT and mutants were overexpressed in HEK293T cells and resolved by SDS–PAGE. The upper part of the membrane, corresponding to the DAPK2 dimer size, was reacted with anti-FLAG antibody diluted 1:1000, and the lower part of the membrane, corresponding to the DAPK2 monomer size, was reacted with anti-FLAG 1:500,000. A representative immunoblot of three independent experiments is shown. **c** HEK293T cells were transfected with different combinations of L1 and L2-fused DAPK2 WT and mutants, and luminescence was measured. Bar graph represents dimerization level as mean ± SD of three technical repeats. Statistical analyses were performed using one-way ANOVA with post hoc Dunnett’s multiple comparison test. ****P < 0.0001 indicates significant decrease in dimerization of all DAPK2 pairs containing a S289D/S289E mutant compared to WT. **d** Expression of the different constructs was assessed using anti-gaussia luciferase antibody that detects both L1 and L2 on western blot. **e** FLAG-DAPK2 WT, S289A or S289D was incubated with MLC in a kinase reaction mixture containing ^32^P-labeled ATP. Reactions were resolved by SDS–PAGE and exposed to X-ray film. pMLC band intensity was quantified using NIH ImageJ software

Aside from removing the auto-inhibitory domain from the catalytic cleft, CaM binding also activates DAPK2 by reducing homodimerization^39^ and decreasing the inhibitory autophosphorylation of Ser308^12^. As Ser289 phosphorylation appears to be an alternative mechanism to CaM binding, we tested its effect on these two properties of DAPK2. When separated by SDS-PAGE, a band at the size corresponding to the DAPK2 homodimer (around 90 kDa) can be detected, representing a dimer fraction that is resistant to the denaturing conditions of the SDS–PAGE^12^. Interestingly, the dimer was less abundant for the Ser289 phospho-mimicking mutants (S289D & S289E), but not the phospho-silencing mutant S289A, compared to WT DAPK2 (Fig. 5a, b), suggesting that the Ser289 phospho-mimicking mutants may have a reduced ability to homodimerize. In order to test this possibility in a quantitative manner, a Protein Complementation Assay (PCA)-based dimerization assay was performed. In this method, two proteins of interest are fused to complementary fragments of the luciferase reporter (referred to as L1 and L2) and luciferase activity is reconstituted upon direct binding of the proteins (reviewed in^40^). HEK293T cells were transfected with different combinations of L1 and L2-fused DAPK2 WT and Ser289 mutants. The luminescence signal represents dimerization of L1 and L2-fused DAPK2 monomers. The two Ser289 phospho-mimicking mutants yielded a significantly reduced signal in all combinations (mutant/mutant or WT/mutant), while the phospho-silencing mutant gave rise to luciferase complementation signals similar to the WT protein (Fig. 5c). Expression levels of the different DAPK2 constructs were assessed by western blot (Fig. 5d). These results indicate that phosphorylation of Ser289 reduces DAPK2 homodimerization.

To assess the effect of Ser289 phosphorylation on DAPK2 autophosphorylation, a radioactive in vitro kinase assay was performed, as Ser308 is a single autophosphorylation site of DAPK2 in vitro^12^. MLC was added as substrate in order to monitor the catalytic activity of the different DAPK2 constructs. Remarkably, autophosphorylation of the S289D mutant was greatly reduced compared to DAPK2 WT and S289A, despite higher catalytic activity towards MLC (Fig. 5e). Thus, the phospho-mimicking mutation of Ser289 strongly reduces DAPK2’s ability to autophosphorylate Ser308. This result provides insight as to the structural effect of Ser289 phosphorylation, as it suggests that similar to CaM binding, Ser289 phosphorylation shifts the inhibitory auto-regulatory domain, containing Ser308, away from the catalytic cleft, thus limiting its phosphorylation. Overall, these results indicate that Ser289 phosphorylation mimics the effects of CaM binding on homodimerization and Ser308 autophosphorylation.

### DAPK2 phosphorylates Beclin-1, inducing Bcl-X_L_ dissociation

**Fig. 6:**
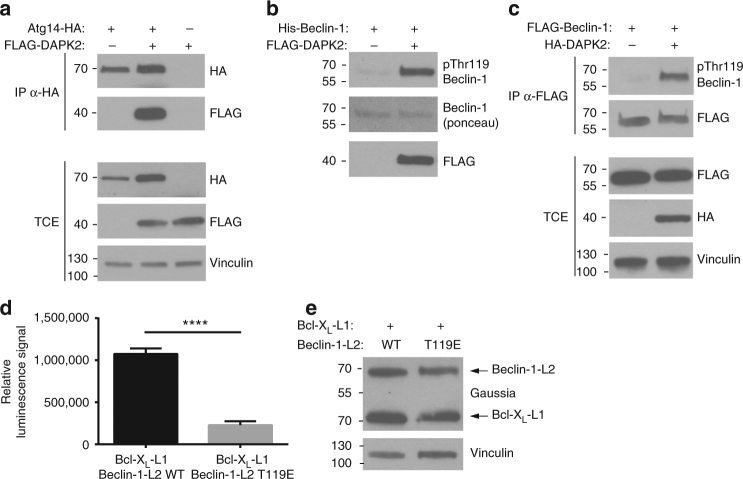
DAPK2 binds Atg14 and phosphorylates Beclin-1, leading to its dissociation from Bcl-X_L_. **a** HEK293T cells were transfected with Atg14-HA and FLAG-DAPK2. Anti-HA immunoprecipitates were resolved by SDS-PAGE and reacted with the indicated antibodies. Representative immunoblots of 3 independent experiments are shown. **b** FLAG-DAPK2 was incubated with His-Beclin-1 in a kinase reaction mixture. Representative immunoblots of 3 independent experiments are shown. **c** HEK293T cells were transfected with FLAG-Beclin-1 and HA-DAPK2. Anti-FLAG immunoprecipitates were resolved by SDS–PAGE and reacted with the indicated antibodies. Representative immunoblots of three independent experiments are shown. **d** HEK293T cells were transfected with BCL-X_L_-L1 and either Beclin-1-L2 WT or T119E. Cells were lysed and luminescence was measured. Bar graph represents binding level as mean ± SD of three technical repeats. Statistical analyses were performed using unpaired two-tailed Student’s *t*-test. ****P < 0.0001. **e** Western blot of **d**

In a screen for protein-protein interactions performed in our lab using the PCA method^21^, DAPK2 was found to specifically interact with Atg14, a member of the Beclin-1 complex involved in autophagy initiation^5,6^. To further validate the binding between DAPK2 and Atg14, a co-immunoprecipitation experiment was performed. HEK293T cells were transfected with FLAG–DAPK2 and Atg14-HA or empty vector as control. Indeed, DAPK2 was co-immunoprecipitated with Atg14, but not with the control (Fig. 6a).

Notably, binding of different kinases to Atg14 was shown to mediate their ability to phosphorylate Beclin-1^41-43^. Moreover, DAPK1, a close family member of DAPK2, can phosphorylate Beclin-1 on Thr119^44^. This phosphorylation, which lies within the BH3 domain of Beclin-1, leads to dissociation of Beclin-1 from its inhibitor, Bcl-X_L_, thus promoting autophagy induction^44,45^. To test if DAPK2 could also directly phosphorylate Beclin-1 on Thr119, an in vitro kinase assay using FLAG-DAPK2 and recombinant human His-Beclin-1 was performed. Beclin-1 phosphorylation was monitored using an antibody generated to specifically recognize Beclin-1 Thr119 phosphorylation. Indeed, DAPK2 could directly phosphorylate Beclin-1 on Thr119 in vitro (Fig. 6b). Furthermore, co-expression of DAPK2 and Beclin-1 in HEK293T cells significantly induced Beclin-1 Thr119 phosphorylation (Fig. 6c), suggesting that Beclin-1 is a bona fide substrate of DAPK2 in cells.

Next, the effect of Thr119 phosphorylation on the Beclin-1/Bcl-X_L_ interaction was monitored using the PCA method, which allows quantitative measuring of protein–protein interactions directly, without the use of antibodies. HEK293T cells were transfected with Bcl-X_L_-L1 and either Beclin-1-L2 WT or T119E. Indeed, the T119E mutant resulted in a significantly lower (~5 fold decrease) luminescence signal compared to Beclin-1 WT (Fig. 6d). Equal expression was verified by western blot (Fig. 6e). These results concur with previous co-immunoprecipitation experiments^44,45^, and confirm that phosphorylation of Beclin-1 on Thr119 promotes its dissociation from Bcl-X_L_, an event that is known to promote autophagy induction^7^.

### The AMPK-DAPK2 axis promotes Beclin-1 Thr119 phosphorylation

**Fig. 7:**
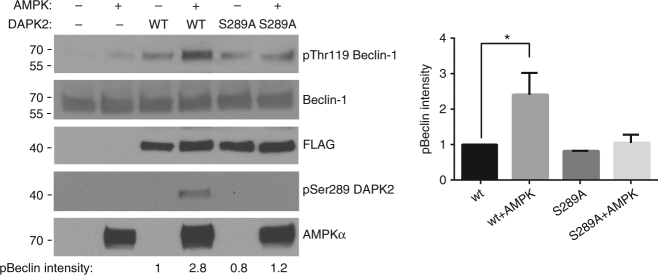
Ser289 phosphorylation enhances DAPK2’s ability to phosphorylate Beclin-1. Sequential kinase assays of AMPK on FLAG-DAPK2 in AMPK kinase buffer, and then of DAPK2 on Beclin-1 in DAPK2 kinase assay buffer. Phosphorylation was assessed by western blotting of the reaction mixtures. Bar graph represents pBeclin-1 intensity as mean ± SD of two independent repeats. Statistical analyses were performed using one-way ANOVA with post hoc Dunnett’s multiple comparison test. *P < 0.05

Next, we tested if phosphorylation of DAPK2 on Ser289 could enhance DAPK2’s ability to phosphorylate Beclin-1. FLAG-DAPK2 immunoprecipitated from HEK293T cells was incubated with recombinant AMPK in a kinase reaction mix containing AMPK kinase assay buffer, thus allowing AMPK to phosphorylate DAPK2. Next, the mix was diluted with DAPK2 kinase assay buffer and recombinant Beclin-1 was added. As a control, the same reaction was carried out using a DAPK2 S289A mutant that cannot be phosphorylated by AMPK. Beclin-1 phosphorylation was significantly higher when WT DAPK2 was pre-incubated with AMPK and phosphorylated by it on Ser289 (Fig. 7). However, pre-incubation of DAPK2 S289A with AMPK did not enhance Beclin-1 phosphorylation. Hence, phosphorylation of DAPK2 on Ser289 enhances its catalytic activity towards Beclin-1. These results suggest that the AMPK–DAPK2 axis promotes Beclin-1 Thr119 phosphorylation and thus contributes to autophagy induction in response to metabolic stress.

### DAPK2 mediates autophagy induction following AMPK activation

**Fig. 8:**
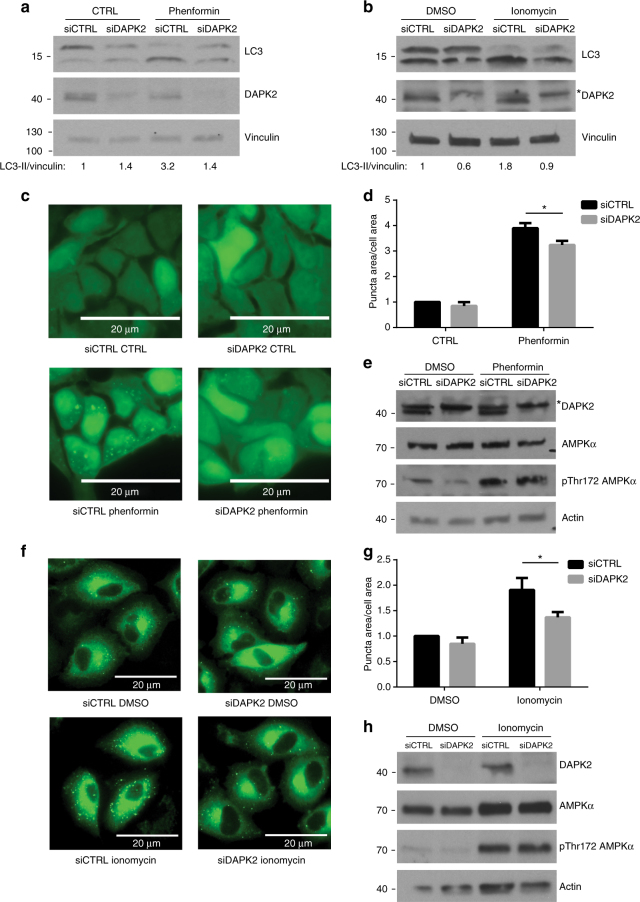
DAPK2 mediates autophagy induction in response to AMPK activation. **a** HCT116 cells were transfected with siRNA targeting DAPK2 or with non-targeting siRNA and treated with 5 mM phenformin for 4 h or left untreated as control. Representative immunoblots of 3 independent experiments are shown. **b** A549 cells were transfected with siRNA targeting DAPK2 or with non-targeting siRNA and treated with 10μM ionomycin or DMSO for 1 h. *Non-specific band. Representative immunoblots of three independent experiments are shown. **c** HEK293 GFP-LC3B cells were transfected with siRNA targeting DAPK2 or with non-targeting siRNA and treated with 5 mM phenformin for 2 h or left untreated as control. Cells were fixed and imaged and puncta area/cell area was measured using MetaMorph software. Scale bar=20 µm. Additional images are shown in Supplementary Figure 4. **d** Bar graph represents puncta area/cell area as mean ± SD of three biological repeats. Statistical analyses were performed using unpaired two-tailed Student’s *t*-test. *P < 0.05. **e** Western blot of a representative experiment. *Non-specific band. **f** A549-DFCP1-GFP cells were transfected with siRNA targeting DAPK2 or with non-targeting siRNA and treated with 10 μM ionomycin or DMSO for 1 h. Cells were fixed and imaged and puncta area/cell area was measured using MetaMorph software. Scale bar=20 µm. Additional images are shown in Supplementary Figure 5. **g** Bar graph represents puncta area/cell area as mean ± SD of three biological repeats. Statistical analyses were performed using unpaired two-tailed Student’s *t*-test. *P < 0.05. **h** Western blot of a representative experiment

To determine if DAPK2 is necessary for autophagy induction in response to metabolic stress, HCT116 cells were transfected with siRNA targeting DAPK2, or with non-targeting siRNA, and treated with phenformin or left untreated as control. Phenformin led to the induction of autophagy, as reflected by the lipidation of LC3 (LC3-II). Notably, depletion of DAPK2 reduced the levels of lipidated LC3 (Fig. 8a), indicating that DAPK2 is required for autophagy induction in response to AMPK activation. This result was further validated in ionomycin-treated A549 cells transfected with DAPK2 siRNA (Fig. 8b). In order to better quantify the level of autophagy induction, HEK293 cells stably expressing GFP-LC3B were transfected with siRNA targeting DAPK2, or with non-targeting siRNA, and treated with phenformin or left untreated as control. Phenformin treatment induced a strong elevation in the number of GFP-LC3B puncta, and depletion of DAPK2 reduced the number of GFP-LC3B puncta (Fig. 8c–e). This result was also validated in ionomycin-treated A549 cells. A549 cells stably expressing DFCP1-GFP were transfected with siRNA targeting DAPK2, or with non-targeting siRNA, and treated with ionomycin or with DMSO as control. DFCP1 is a PI(3)P binding protein that marks the precursor structures that lead to formation of autophagosomes^46^. Therefore, the amount of DFCP1-GFP puncta specifically indicates the level of Beclin-1/Atg14/Vps34 complex activity and autophagy induction. While ionomycin induced a strong elevation in the number of DFCP1-GFP puncta, depletion of DAPK2 reduced the number of DFCP1-GFP puncta (Fig. 8f–h). Altogether, these results suggest that DAPK2 is an important downstream effector of AMPK in promoting autophagy.

## Discussion

As DAPK2 is involved in several critical cellular processes, including autophagy, its activity in cells is tightly regulated. The two known regulatory phosphorylations of DAPK2 (autophosphorylation on Ser308 and trans-phosphorylation on the C-terminal tail) are inhibitory. In this work, we have identified an activating phosphorylation event: phosphorylation of Ser289 by AMPK. We have confirmed that this phosphorylation occurs in response to AMPK activation in cells. Moreover, it occurs in vivo in mouse muscle tissues as part of the physiological response to fasting. This is the first time that DAPK2 was shown to be activated by metabolic stress. It should be noted, that a human protein microarray screen for cGK-I substrates identified DAPK2, and one of the phosphorylated residues found was Ser289^47^. It is possible that Ser289 can be phosphorylated by several kinases. However, as phosphorylation by cGK-I was observed only in vitro, further work needs to be done in order to determine if it is indeed a regulator of DAPK2 in cells.

DAPK2 activity is regulated by several inhibitory mechanisms. Until now, it was assumed that DAPK2 must undergo Ser308 dephosphorylation and bind CaM in order to become fully active. Interestingly, Ser289 phosphorylation activates DAPK2 without either Ser308 dephosphorylation, as observed using the S289D S308D double mutant, or CaM binding. Thus, Ser289 phosphorylation provides a novel, alternative mechanism for DAPK2 activation, independent of calcium signaling. We found that Ser289 phosphorylation functionally mimics two effects of CaM binding: reduction in Ser308 autophosphorylation and decreased homodimerization. Remarkably, Ser289 phosphorylation not only mimics the effects of CaM binding, but also reduces DAPK2’s affinity to CaM, thus comprising a completely separate regulatory pathway.

**Fig. 9:**
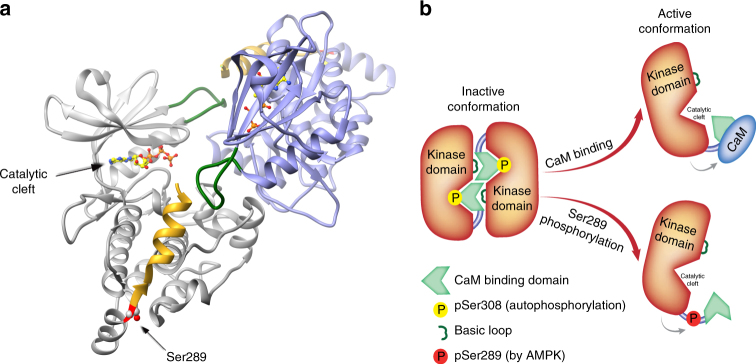
Ser289 resides in a critical position in the DAPK2 structure. **a** Ribbon diagram of the DAPK2 dimer (PDB code 2A2A^11^). The two monomers are shown in gray and purple. The C-terminal part (residues 290–304), containing part of the CaM binding helix, is marked in gold, the basic loop (residues 46–56) is in dark green and Ser289 is in red (marked with an arrow). An ATP molecule marks the position of the catalytic cleft. **b** Proposed mechanism of activation by Ser289 phosphorylation. In the inactive state, the CaM binding domain (in light green) blocks the catalytic cleft, Ser308 is autophosphorylated, and DAPK2 is dimeric. Dimerization is mediated in part by interaction of the basic loop (in dark green) with the CaM auto-regulatory domain of the other monomer. CaM binding or Ser289 phosphorylation activate DAPK2 by shifting the CaM binding domain away from the kinase domain, thus exposing the catalytic cleft, reducing Ser308 autophosphorylation and decreasing dimerization. The C-terminal tail of DAPK2 is not represented in this scheme since it is lacking from the available crystal structures

According to the published crystal structure of DAPK2 (1–301)^11^, Ser289 is located in a short loop (residues 289–292) right before the inhibitory CaM binding helix (residues 302–320. See Fig. 9a). This loop was proposed to act as a flexible hinge that allows a swing-out motion of the CaM binding helix^31^. Therefore, a possible explanation for the multiple effects of Ser289 phosphorylation is that phosphorylation at this critical site causes a conformational change in the protein structure that alters the positioning of the following CaM binding helix. The fact that Ser289 phosphorylation considerably reduces DAPK2’s ability to autophosphorylate Ser308, which resides within this helix, supports this hypothesis. This proposed conformational change can explain how a single phosphorylation affects several different properties of DAPK2. Homodimerization of DAPK2 is mediated in part by an interaction between the CaM auto-regulatory domain of one monomer and the basic loop in the kinase domain of the other monomer^11,39^. Likewise, CaM binding is mediated by initial low-affinity binding to the basic loop, leading to high-affinity binding to the CaM auto-regulatory domain^39^. As the suggested conformational change conferred by Ser289 phosphorylation shifts the CaM auto-regulatory domain away from the kinase domain, both homodimerization and CaM binding would be affected (see scheme in Fig. 9b). Catalytic activity would be enhanced by the removal of the CaM auto-regulatory domain away from the catalytic cleft, as well as by the reduction in Ser308 autophosphorylation and homodimerization.

In this work, we have identified a new substrate of DAPK2, Beclin-1. Specifically, we have shown that DAPK2 phosphorylates Thr119 within the BH3 domain of Beclin-1, thus promoting dissociation of Beclin-1 from its inhibitor Bcl-X_L_, a key step in autophagy induction. Even though it has been quite some time since DAPK2 was first implicated in autophagy induction^18^, this is the first core autophagic machinery protein that was found to be a direct substrate of DAPK2. As very few substrates of DAPK2 have been identified so far, this finding is of additional significance. Interestingly, the same site on Beclin-1 was found to be phosphorylated by two additional kinases, DAPK1^44^ and ROCK^45^, highlighting its importance as a hotspot in the regulation of the Beclin-1-Bcl-X_L_ interaction.

The similar substrate preference of DAPK2 and DAPK1 stems from the ~80% sequence identity shared by their kinase domains. The CaM auto-regulatory domains are slightly less conserved, at 50% sequence identity. Although Ser289 is conserved in DAPK1, it is not phosphorylated upon metabolic stress-induced AMPK activation, but it is phosphorylated upon activation of the opposing MAPK/ERK survival signaling pathway. Moreover, in the context of cell surviving signaling, Ser289 phosphorylation of DAPK1 conversely inhibits its apoptotic activity in cells^38^. These major differences between the two kinases can be explained by the fact that DAPK1 and DAPK2 differ substantially outside of their kinase and CaM auto-regulatory domains^9^. DAPK1 (160 kDa) has many extra-catalytic regulatory and protein interacting domains that DAPK2 (42 kDa) lacks, such as a ROC-COR domain and a death domain. In contrast, DAPK2 has a unique 40 amino acid tail that was shown to have important distinct regulatory functions. Thus, although DAPK1 and DAPK2 may possess similar substrate preferences, such as Beclin-1, their regulation in cells by upstream modulators, and the spectrum of their interacting proteins, is expected to be quite different. Here, we show for the first time differential regulation of the two kinases by opposing triggers. These profound differences in the regulation of DAPK1 and DAPK2 are of great interest, and shed some light on the different roles that these similar kinases may serve in cells.

We have shown by siRNA knock-down experiments that depletion of DAPK2 reduces autophagy induction in response to AMPK activation. This indicates that DAPK2 is one of AMPK’s important downstream targets in promoting autophagy. Specifically, it suggests that DAPK2 Ser289 phosphorylation enhances its pro-autophagic activity in cells. Accordingly, in vitro phosphorylation of DAPK2 by AMPK enhanced DAPK2’s ability to phosphorylate Beclin-1. Interestingly, AMPK phosphorylates Beclin-1 directly on several sites outside its BH3 domain^43,48^. Moreover, activation of AMPK by metformin promotes Beclin-1-Bcl-2 dissociation, through activation of JNK-mediated phosphorylation of Bcl-2^49^. The fact that AMPK promotes Beclin-1 activation through several different mechanisms, by direct phosphorylation as well as by activating other kinases, emphasizes the importance of this step in autophagy initiation.

Interestingly, Beclin-1 is not the only shared target of AMPK and DAPK2. An additional shared substrate of AMPK and DAPK2 is the mTOR binding protein raptor. AMPK inhibits mTOR through direct phosphorylation of raptor on Ser722 and Ser792^50^, and DAPK2 phosphorylates raptor on Ser721^19^, also contributing to mTOR inhibition. The fact that AMPK and DAPK2 share several mutual pathways and substrates supports a functional link between the two kinases, highlighting the significance of the AMPK-DAPK2 pathway.

## Methods

### Cell culture and induction of cell stress

HEK293T cells, A549, and HCT116 cells were grown in Dulbecco’s Modified Eagle’s medium (Biological Industries, Beit Haemek, Israel), supplemented with 10% fetal bovine serum (FBS, GibcoBRL), 4 mM glutamine (GibcoBRL), and combined antibiotics (100 µg/ml penicillin and 0.1 mg/ml streptomycin). HEK293 GFP-LC3B and A549 DFCP1-GFP were grown also in the presence of 1 mg/ml G418. Cells were treated with the following reagents: DMSO, ionomycin, phenformin, resveratrol (Sigma-Aldrich), compound C (Calbiochem), TRAIL (PeproTech), A-769662 (InvivoGen). All cell lines were purchased from the ATCC and routinely tested for mycoplasma. No cell lines used in this paper were listed in the database of commonly misidentified cell lines maintained by ICLAC.

### DNA constructs

FLAG-tagged DAPK2 WT and different mutants were expressed from pcDNA3 expression vectors. Control plasmid consisted of empty pcDNA3 plasmid. Point mutations were generated using QuikChange protocol (Stratagene). The sequence of the coding region was confirmed by DNA sequencing. For cloning of Bcl-X_L_, Beclin-1, and DAPK2 into the Gaussia luciferase plasmids, a cDNA library was generated from HEK293 cells using RNAeasy MiniElute Cleanup kit (QIAGEN) for mRNA production and SuperScript First-Strand kit (Invitrogen) for RT-PCR. The different genes were amplified with specific primers containing additional restriction sites for BSPEI and XbaI, or NotI and ClaI for the insertion into GLuc(1)-X or X-GLuc(1)/X-GLuc(2) plasmids, respectively. After a standard cloning procedure, the final product was confirmed by direct sequencing of the entire ORF.

### Transfection procedures

HEK293 and HEK293T cells were transfected by standard calcium phosphate technique. A549 were transfected using JetPrime (PolyPlus) for DNA transfection or Lipofectamine2000 (Thermo Fisher Scientific) for siRNA transfection. HCT116 cells were transfected using JetPEI (PolyPlus) for DNA transfection or Dharmafect2 (Dharmacon) for siRNA transfection. All transfections were performed according to the manufacturer’s protocol.

### RNA interference

DAPK2 expression was transiently knocked down in cells using siGENOME SMARTpool siRNA reagent (Dharmacon, Lafayette, CO, USA) against human DAPK2 (NM_014326). For control siRNA, siCONTROL non-targeting siRNA was used (Dharmacon).

### Protein analysis

Cells were lysed in PLB buffer (10 mM NaPO_4_ pH 7.5, 5 mM EDTA, 100 mM NaCl, 1% Triton X-100, 1% Na deoxycholate, 0.1% SDS) or with TNE buffer (1% NP-40, 20 mM Tris–HCl pH 7.5, 150 mM NaCl, 1 mM EDTA), supplemented with 1 μM PMSF and protease and phosphatase inhibitor cocktails (Sigma). Proteins were separated by SDS–PAGE and transferred to nitrocellulose membranes, which were incubated with antibodies against Flag (1:10,000–1:100,000, F3165), LC3B (1:10,000, L7543), α-tubulin (1:200,000, T9026), vinculin (1:200,000, V9131), actin (1:10,000, A3853), ERK1/2 (1:100,000, M5670), active diphosphorylated (Thr183/Tyr185) ERK1/2 (1:5000, M8159) (all from Sigma), HA (1:10,000-1:50,000, 901502, BioLegend), DAPK2 (1:500, ab51601, Abcam), Gaussia Luciferase (1:1000, #401, NanoLight), MLC (1:10,000, #3672), phosphoSer19-MLC (1:500, #3671), AMPK (1:1000, #2603), phospho Thr172-AMPK (1:1000, #2535), phospho-Akt substrate (1:1000, RXRXXS*/T*) (C-10001), cleaved caspase 3 (1:1000, C-9664), cleaved caspase 9 (1:500, #9501) (all from Cell Signaling), PARP-1 (1:5000, BML-SA250, Biomol), or Beclin-1 (1:1000, sc-48341, Santa Cruz). Polyclonal antiphospho-Ser289 DAPK2 antibody was raised in rabbits immunized with the phosphorylated peptide CMVRRE(pS)VVNLEN (Bethyl Laboratories), which is 100% identical in human and mouse DAPK2. Polyclonal antiphospho-Thr119 Beclin-1 antibody was raised in rabbits immunized with the phosphorylated peptide CRLKV(pT)GDLF (Bethyl Laboratories). Detection was done with either HRP-conjugated goat anti-mouse or anti-rabbit secondary antibodies (Jackson ImmunoResearch), followed by enhanced chemiluminescence (EZ-ECL, Biological Industries Israel Beit-Haemek Ltd.). Protein densitometric analysis was performed using ImageJ (NIH Imaging Software) on scanned blots. Uncropped images of several important immunoblots are presented in Supplementary Figure 7.

### Immunoprecipitation

Protein extracts were incubated with anti-FLAG M2 beads or anti-HA beads (Sigma-Aldrich). After multiple washes, the immunoprecipitated protein was eluted from the beads with an excess of FLAG or HA peptides (Sigma-Aldrich).

### Kinase assays

DAPK2 kinase assay: 30 ng FLAG-DAPK2 immunoprecipitated from HEK293T cells was incubated with 2 µg MLC or 750 ng Beclin-1 (ProSpec) in DAPK2 kinase assay buffer (50 mM Hepes pH 7.5, 20 mM MgCl_2_, 50 µM ATP, protease and phosphatase inhibitor mix (Sigma), 500 mM EGTA or 0.5 mM Ca^2+^, 1 µM CaM) for 5 min (MLC) or 15 min (Beclin-1) at 30 °C. Reactions were stopped with sample buffer and resolved by SDS–PAGE.

AMPK kinase assay: For radioactive kinase assay, 1 µg DAPK2 K42A or 1 µg DAPK2 K42A S289A purified from *E. coli* was incubated with 500 ng recombinant AMPK (α1/β1/γ1) (Sigma) in kinase assay buffer (5 mM MOPS, 1 mM EGTA, 0.4 mM EDTA, 5 mM MgCl_2_, 0.05 mM DTT, 50 µM cold ATP, 5µCi [γ-^32^P]ATP, 100 µM AMP, protease and phosphatase inhibitor mix) for 30 min at 30 °C. Reactions were stopped with sample buffer and resolved by SDS–PAGE. Gels were dried and radioactive signal was detected using Amersham Hyperfilm and ^32^P intensifying screen (Kodak). For cold kinase assay, 1 µg DAPK2 K42A or 1 µg DAPK2 K42A S289A was incubated with 200 ng recombinant AMPK in kinase assay buffer for 30 min at 30 °C and reactions resolved by SDS–PAGE for western blotting.

AMPK/DAPK2 double kinase assay: 75 ng DAPK2 was incubated with 100 ng recombinant AMPK (α1/β1/γ1) (Sigma) in AMPK kinase assay buffer (for 30 min at 30 °C). Next, 40% of the reaction volume was diluted in DAPK2 kinase assay buffer containing 400 ng MLC or 1 µg Beclin-1, and incubated for 5 min or 10 min, respectively, as described above.

### Animals

Animal experiments were consistent with the National Israeli guidelines for the care and use of laboratory animals, and were approved by the Technion Inspection Committee on the Constitution of the Animal Experimentation. Adult male CD-1 mice (27–28 g, about 8 weeks old) were purchased from Envigo RMS (Israel), and animal care was provided by specialized personnel in the institutional animal care facility. In fasting experiments, mice were deprived of food for 24 or 48 h (water was available ad libitum), and following euthanasia, tibialis anterior muscles were excised and stored at −80 °C.

### Skeletal muscle homogenization and measurements

Mouse tibialis anterior muscles were homogenized and lysed in homogenization buffer (20 mM Tris–HCl, pH 7.2, 5 mM EGTA, 100 mM KCl, 1% Triton X-100, 2 mM sodium orthovanadate, 10 mM sodium pyrophosphate, 50 mM sodium fluoride, 10ug/ml leupeptin, 3 mM benzamidine, 1 mM PMSF and PhosSTOP (Roche). After centrifugation at 6000 × *g*, the supernatant (i.e., cytosolic fraction) was collected.

Muscles excised from fed or fasted mice were snap-frozen in isopentane, and cross-sections were fixed in 4% PFA. Cross-sectional areas of fibers in 10-μm muscle sections were measured using Imaris (Bitplane), and data collected from at least 500 fibers from four fed or fasted mice were plotted. Images were collected using a Nikon Ni-U upright fluorescence microscope with a Plan Fluor 20 Å ~0.5 NA objective lens with a Hamamatsu C8484-03 cooled CCD camera and MetaMorph software.

### Mass spectrometry phosphopeptide analysis

A total of 2 µg DAPK2 K42A was incubated with or without 200 ng AMPK in kinase assay buffer for 10 min at 30 °C. Reactions were stopped with sample buffer and resolved by SDS–PAGE. The gel was stained with GelCode Blue (Pierce), and gel bands excised and subjected to mass spectrometric analysis at the Smoler Proteomics Center at the Technion (Haifa, Israel). The proteins in the gel were reduced with 10 mM DTT, modified with 40 mM iodoacetamide and trypsinized (modified trypsin (Promega)) at a 1:100 enzyme-to-substrate ratio. The resulting tryptic peptides were resolved by reverse-phase chromatography on 0.1 × 200-mm fused silica capillaries (J&W, 100 micrometer ID) packed with Everest reversed phase material (Grace Vydac, CA). The peptides were eluted with linear 120 min gradients of 5–95% of acetonitrile with 0.1% formic acid in water at flow rates of 0.4 µl/min. Mass spectrometry was performed by an ion-trap mass spectrometer (Orbitrap, Thermo) in a positive mode using repetitively full MS scans followed by collision induced dissociation (CID) of the 5 most dominant ions selected from the first MS scan. The mass spectrometric data was clustered and analyzed using the Sequest software and Pep-Miner, searching against the human sequences within the NR-NCBI database.

### Protein complementation assay

Fragments of the Guassia luciferase (referred to as L1 (amino acids 1–93) and L2 (amino acids 94–169)) were fused to the indicated proteins and transfected in pairs into HEK293T cells. Cell lysis and luminescent readings were performed, as described in ref. ^21^.

### ELISA CaM binding assay

96-well ELISA MaxiSorp plates (Nunc) were coated with 1 µg CaM (Sigma P2277) and incubated at 4 °C overnight. After washing in PBST and blocking in blocking solution (4% BSA, 0.02% azide in PBS), wells were incubated with 80 ng DAPK2 for 15 min at 30 °C, washed again and incubated with anti-DAPK2 antibody (Abcam ab51601) followed by HRP-conjugated goat anti-rabbit secondary antibody (Jackson ImmunoResearch). The wells were further incubated with fresh ABTS substrate solution (1.4 ml citric acid 0.2 M, 2.2 ml Na_2_HPO_4_ 0.2 M, 6.4 ml DDW, 50 μl H_2_O_2_ and 10 mg ABTS) and color reaction was quantified using ELISA plate reader. To verify equal loading of the different DAPK2 mutants, wells were coated with the DAPK2 used in the assay, and the amount of DAPK2 was quantified as described above.

### Blebbing assay

HEK293T cells were transfected with GFP together with FLAG-DAPK2 WT or mutants. After 24 h, the cells were imaged by fluorescence microscopy (Olympus BX41). For every transfection, ~250 GFP-positive cells were counted from three separate fields and marked as “blebbed” or “not blebbed”.

### LC3B-GFP and DFCP1-GFP punctate assay

HEK293 cells stably expressing GFP-LC3B were plated onto 18 mm glass coverslips coated with poly-L-Lysine (Sigma-Aldrich) and treated with 5 mM phenformin for 2 h or left untreated as control. A549 cells stably expressing DFCP1-GFP were plated onto 18 mm glass coverslips and treated with 10 μM ionomycin or with DMSO as control. For imaging, cells were fixed with 4% PFA and viewed by fluorescent microscopy (Olympus BX41) with x60 (N.A. 1.25) UPlan-Fl oil immersion objective, and digital images obtained with a DP72 CCD camera using CellSens Standard software (Olympus). For HEK293 GFP-LC3B cells, puncta area/cell area was calculated by analyzing 15–20 different fields, each containing ~100 cells, for each transfection. For A549 DFCP1-GFP cells, puncta area/cell area was calculated by analyzing 20 different fields, each containing ~10 cells, for each transfection. The total area of GFP puncta per total cell area of the population of GFP-expressing cells was quantified by extracting the puncta signals using the Top Hat algorithm of MetaMorph (Molecular Device), and calculating the total area of the dots.

### Bacterial plasmid construction and expression

hMLC was cloned into the His-bsSumo (K151) expression vector using Transfer-PCR. An Ala residue was incorporated at the N-terminal part of hMLC in order to facilitate cleavage of hMLC from the His-bdSumo tag. Expression was performed in E coli Bl21(DE3) cells. Protein production was induced by addition of 0.2 mM IPTG at mid-log phase and the cells were grown overnight at 15 °C. Cells were lysed using a cell disrupter (Constant Systems) at 4 °C in buffer A (50 mM Tris pH 8, 0.5 M NaCl, 20 mM Imidazole with EDTA-free protease-inhibitor tablet, 0.5 mg/ml lysozyme, and DNaseI). After centrifugation, the clarified supernatant was passed over a HiTrap chelating HP 5 ml column (GE, Healthecare). 1 ml bdSumo protease, bdSENP1, without His tag was added to the column which was left at 4 °C overnight. The cleaved protein was collected by washing the column with 10 ml buffer A. hMLC was further purified by size-exclusion chromatography (HiLoad_16/60_superdex75 prepgrade, GE Healthcare) equilibrated with 50 mM Tris pH 8, 0.1 M NaCl.

DAPK2 K42A and K42A S289A were cloned into the pET28-TevH expression vector using Transfer-PCR. Plasmids were expressed in Escherichia coli BL21(DE3) cells. Proteins were purified using excess peptide.

### Multiple sequence alignment

Multiple sequence alignment of DAPK2 orthologues was performed using the ClustalOmega algorithm^51^.

Alignment of DAPK1 and DAPK2 was performed using the Protein BLAST algorithm (blast.ncbi.nlm.nih.gov).

### Molecular graphics

Molecular graphics and analyses were performed with the UCSF Chimera package^52^.

To properly locate the ATP molecule in the catalytic cleft, the structure of mouse DAPK2 in complex with ATP (PDB entry 2YAA) was superposed on the structure of the human DAPK2 dimer (PDB code 2A2A^11^), which was not crystalized with ATP. In the final image, only the human DAPK2 dimer is shown, and the ATP molecule is positioned as in the mouse DAPK2 structure.

### Statistical analysis

Statistical analyses were performed using GraphPad Prism software. Values of *P* < 0.05 were considered significant.

### Data availability

The data that support the findings of this study are available from the corresponding author upon reasonable request.

## Electronic supplementary material

Supplementary Information
