# Supplementary material for: Non-canonical activation of DAPK2 by AMPK constitutes a new pathway linking metabolic stress to autophagy
Source: Nat Commun. 2018 May 1;9:1759. doi: 10.1038/s41467-018-03907-4 (PMC5931534; doi:10.1038/s41467-018-03907-4)
Supplement: Supplementary file 1 — Supplementary Information [file 41467_2018_3907_MOESM1_ESM.pdf]

Non-Canonical Activation of DAPK2 by AMPK Constitutes a New Pathway Linking Metabolic Stress to Autophagy

Shiloh et al.

Supplementary Figure 1.

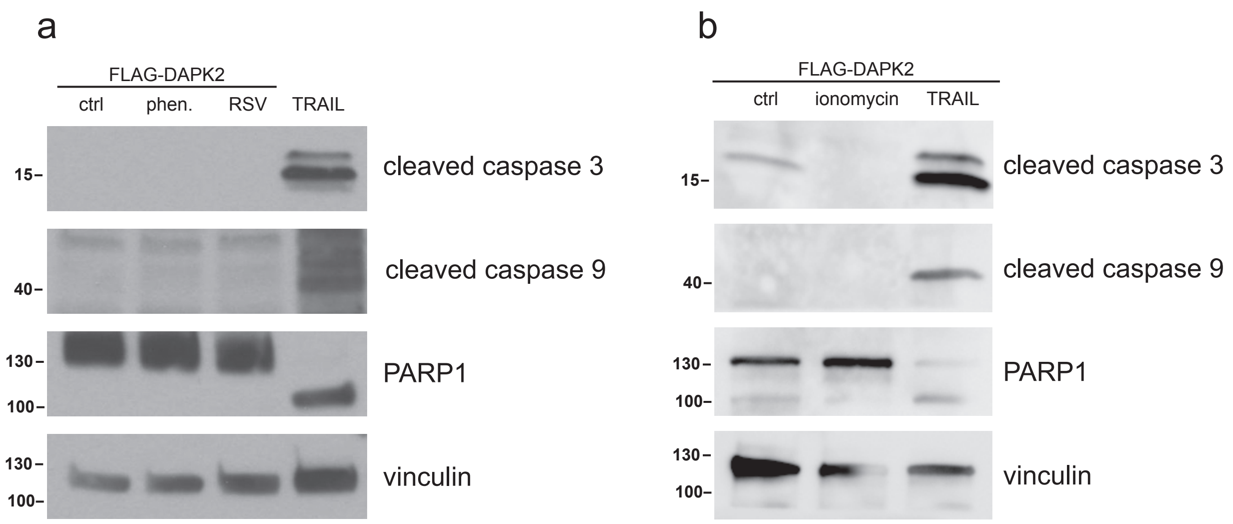

Supplementary Figure 1. AMPK-activating drugs do not induce caspase-dependent apoptosis.

(a) HCT116 cells were transfected with FLAG-DAPK2 WT and treated with either 10mM phenformin for 4h or 200µM resveratrol for 2h and subjected to western blotting for apoptosis markers. A549 cells treated with 100 ng/ml TRAIL and 10µg/ml cyclohexamide were used as a positive control. **(b)** A549 cells were transfected with FLAG-DAPK2 WT and treated with 10µM ionomycin for 1h and subjected to western blotting for apoptosis markers. A549 cells treated with 100ng/ml TRAIL and 10µg/ml cyclohexamide were used as a positive control.

Supplementary Figure 2.

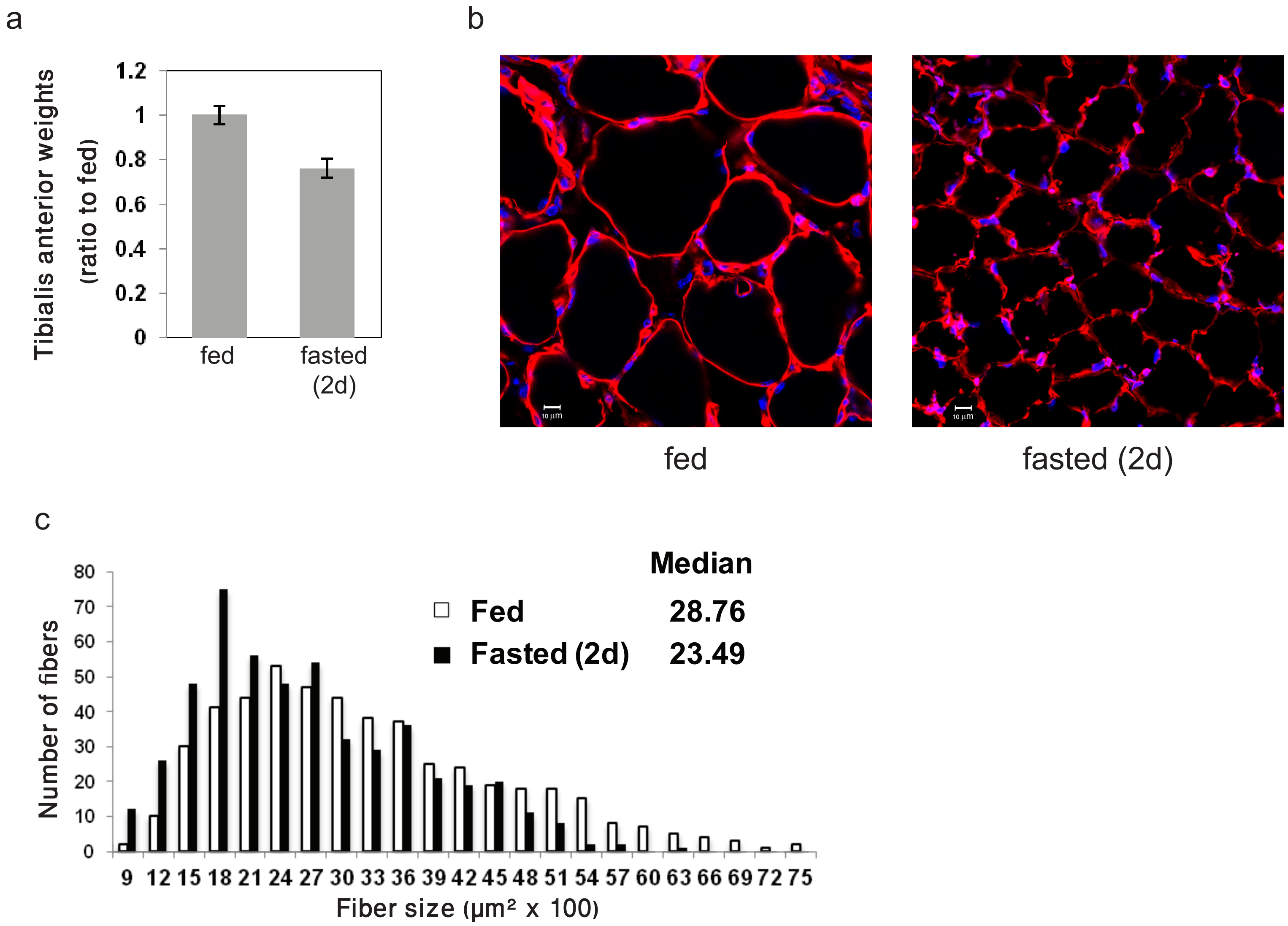

Supplementary Figure 2. 48h of food deprivation causes skeletal muscle atrophy in mice.

(a) Mean weights of Tibialis Anterior muscles from fasted mice are presented as ratio to fed control. Muscles from 6 different mice at each condition were measured. Statistical analyses were performed using paired one-tailed Student's *t*-test. *$P<0.05$. **(b)** Cross sections of Tibialis Anterior muscle from fed and fasted (2 days) mice were fixed and stained for laminin (red). Nuclei were stained using Hoechst 33258 (blue). Scale bar: 10μm. **(c)** Cross-sectional fiber size area of 500 muscle fibers from a total of 4 mice at each condition was measured.

Supplementary Figure 3.

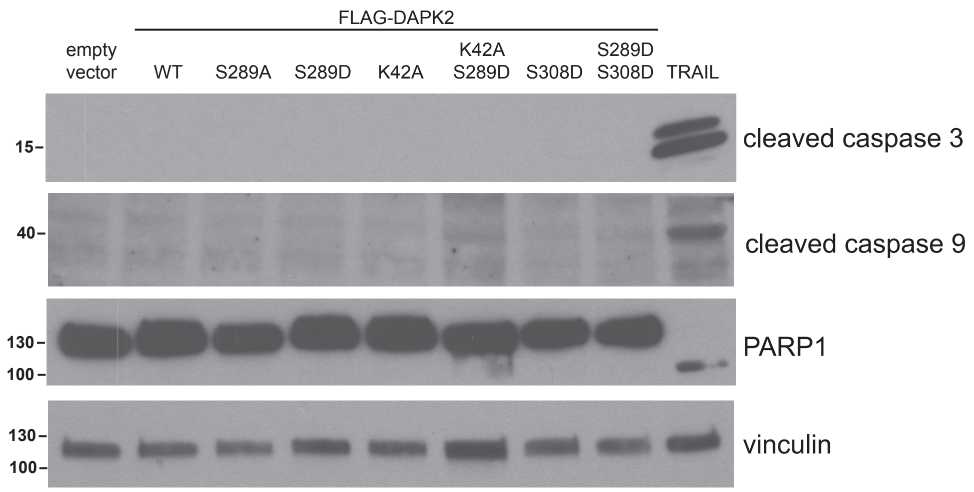

Supplementary Figure 3. Overexpression of DAPK2 WT and different mutants does not induce apoptosis.

293T cells were transfected with DAPK2 WT and different mutants, or empty vector, and subjected to western blotting for apoptosis markers. A549 cells treated with 100ng/ml TRAIL and 10μg/ml cyclohexamide were used as a positive control.

Supplementary Figure 4.

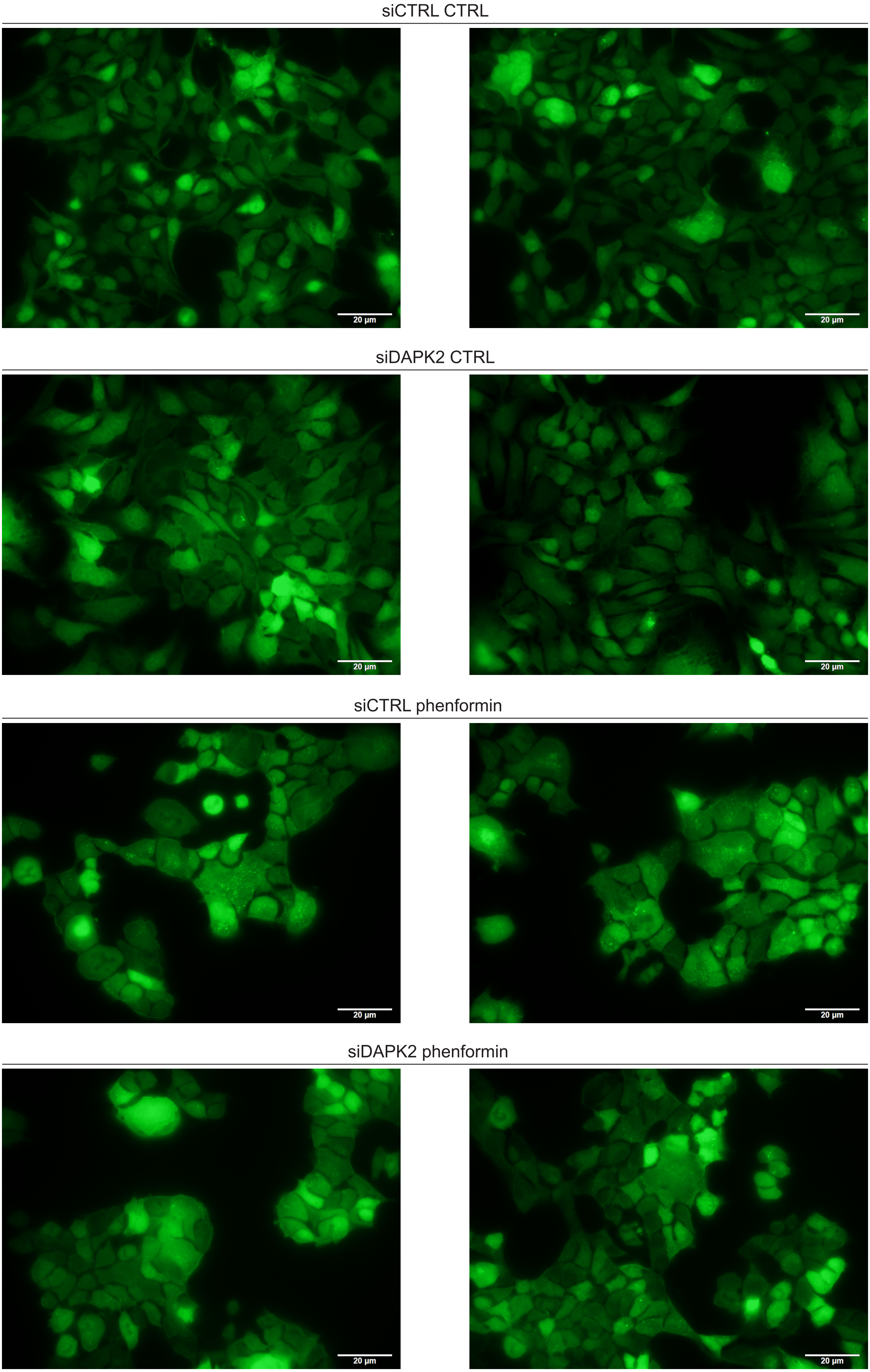

Supplementary Figure 4. Additional images for Figure 8c.

HEK293-GFP-LC3B cells were transfected with siRNA targeting DAPK2 or with non-targeting siRNA and treated with 5mM phenformin for 2h left untreated as control. Cells were fixed and imaged. Scale bar: 20µm.

Supplementary Figure 5.

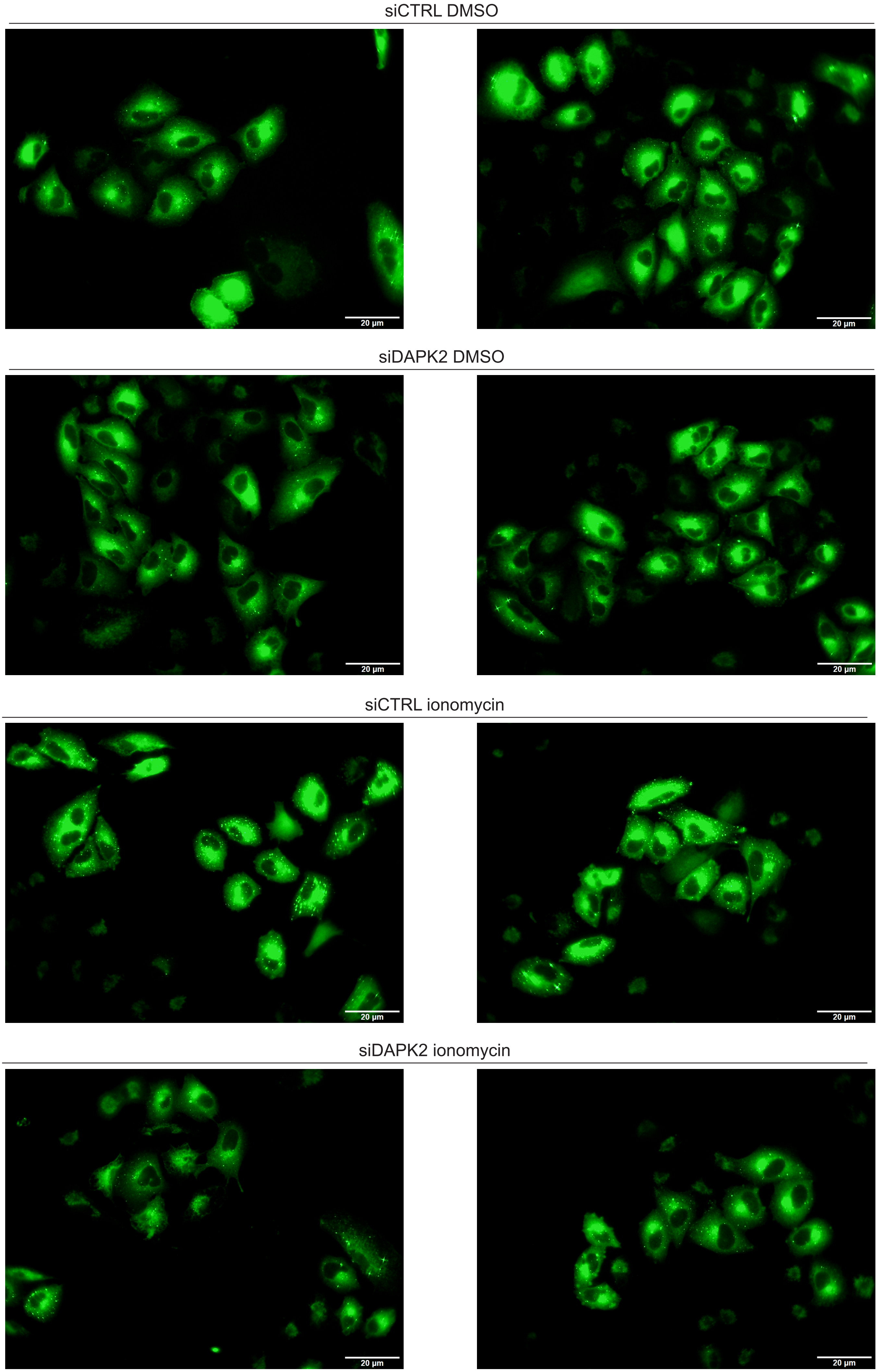

Supplementary Figure 5. Additional images for Figure 8f.

A549-DFCP1-GFP cells were transfected with siRNA targeting DAPK2 or with non-targeting siRNA and treated with 10µM ionomycin or DMSO for 1h. Cells were fixed and imaged. Scale bar: 20µm.

Supplementary Figure 6.

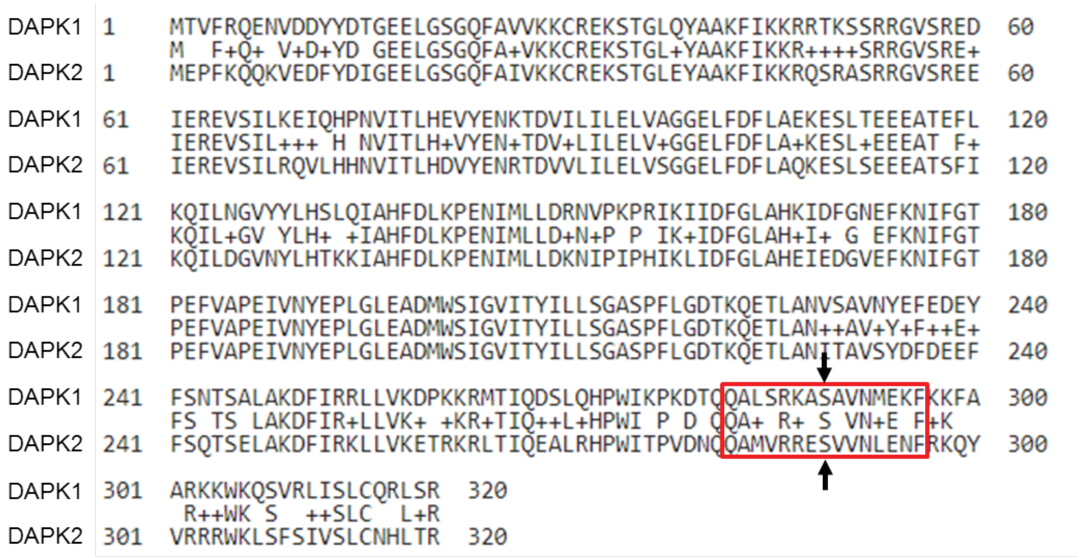

Supplementary Figure 6. Alignment of DAPK1 and DAPK2.

The amino acid sequences spanning the kinase and CaM auto-regulatory domains (1-320) of human DAPK1 (UniProt IP P53355) and human DAPK2 (UniProt ID Q9UIK4) were aligned using the Protein BLAST algorithm. Ser289 is marked with arrows and the sequence surrounding it is highlighted by a red rectangle.

Supplementary Figure 7.

Figure 1c

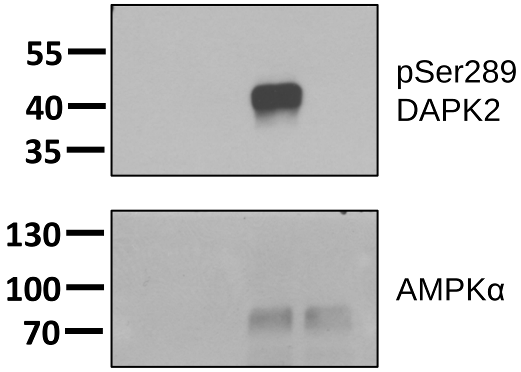

Figure 2a

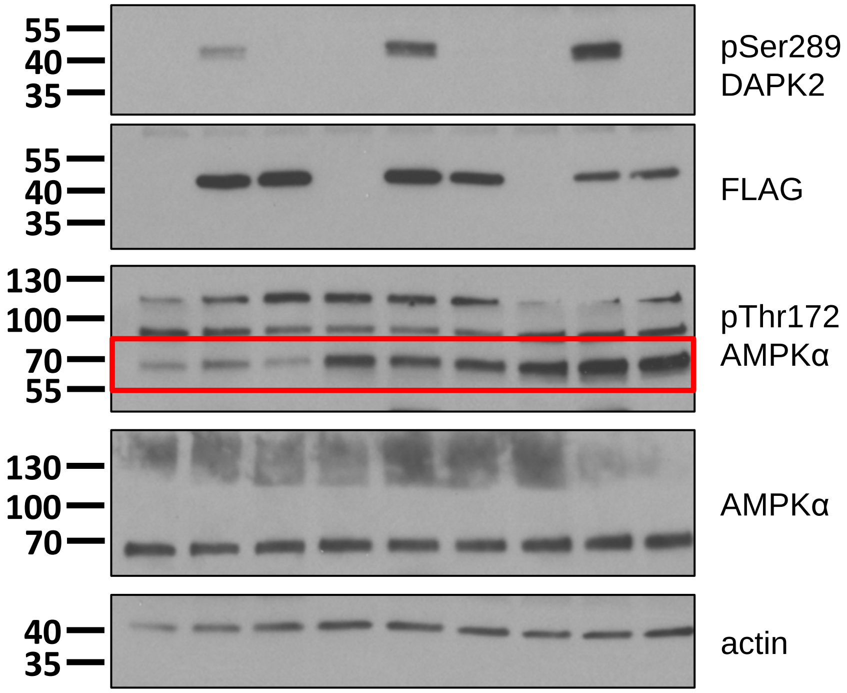

Figure 2b

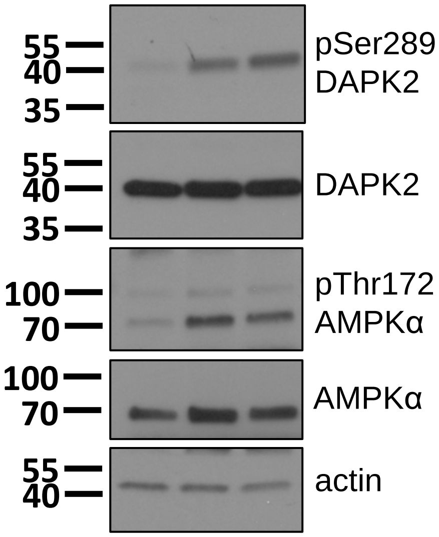

Figure 2c

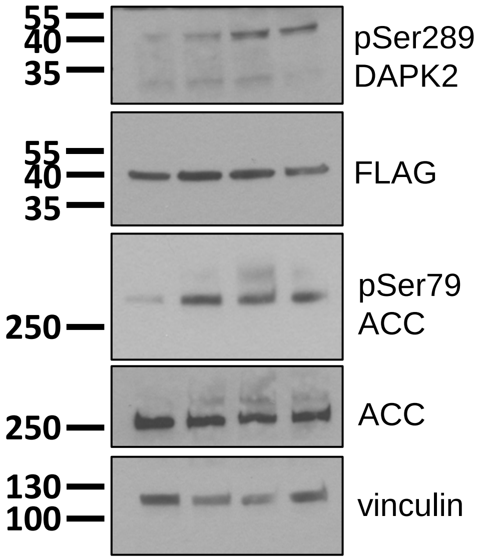

Figure 2d

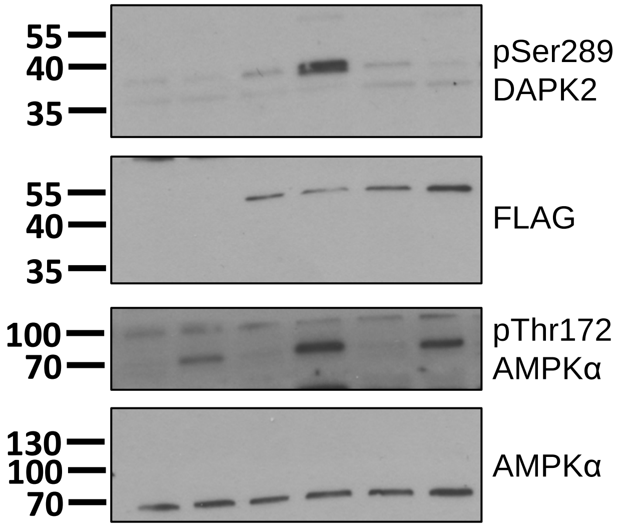

Figure 2e

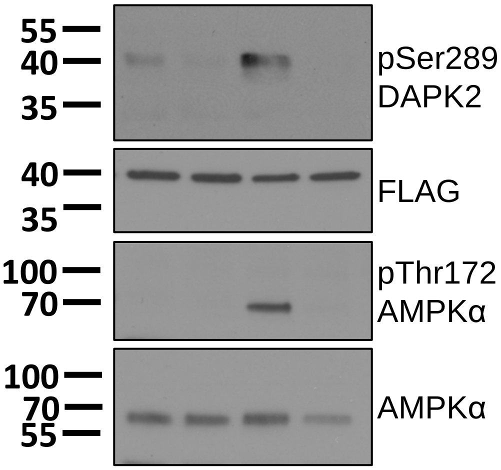

Figure 2f

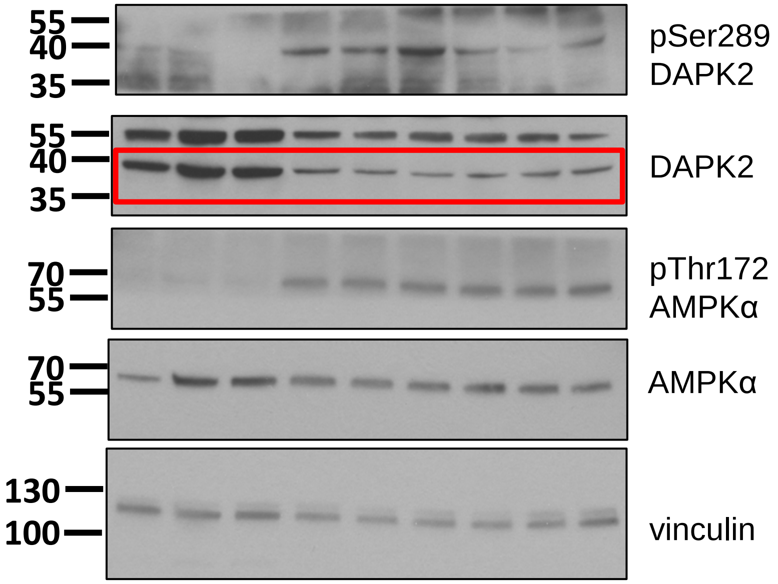

Figure 3a

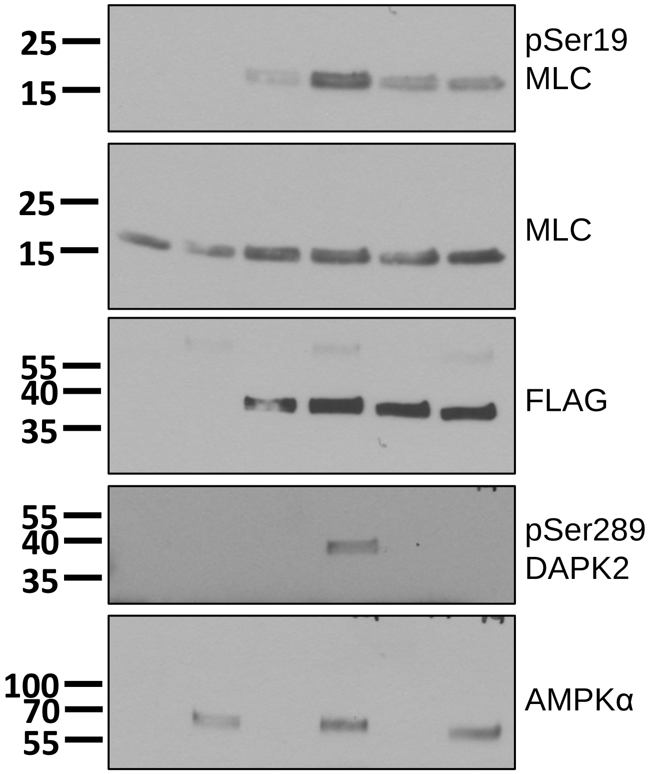

Figure 3b

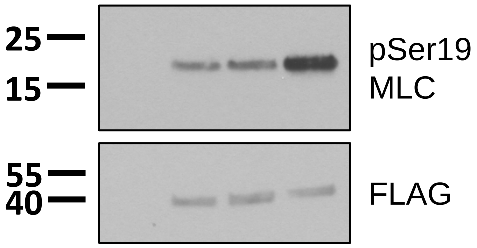

Figure 4a

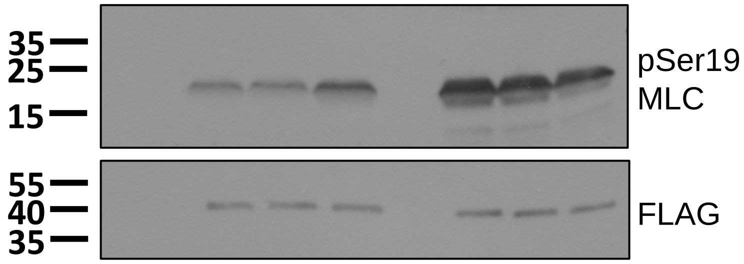

Figure 6b

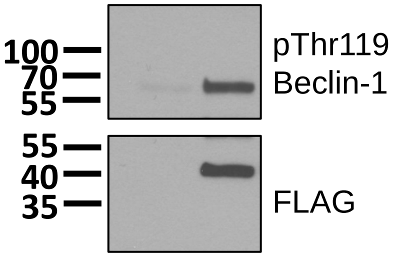

Figure 7

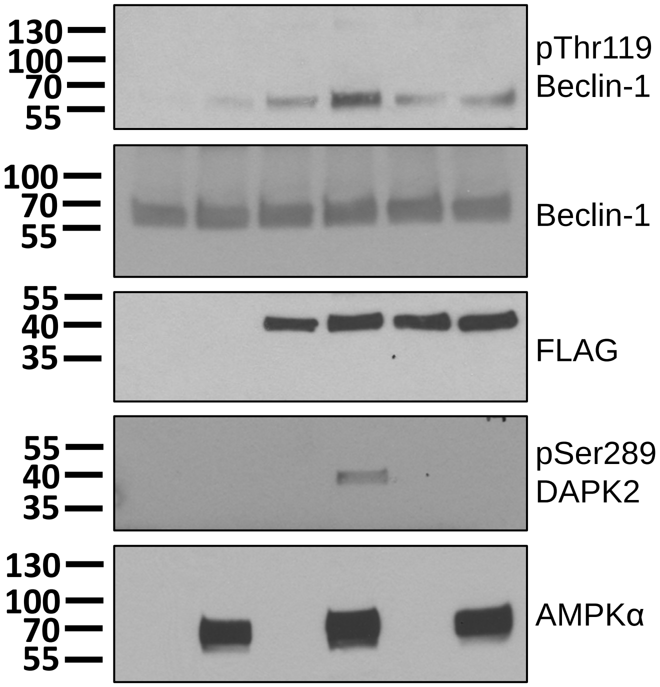

Figure 8a

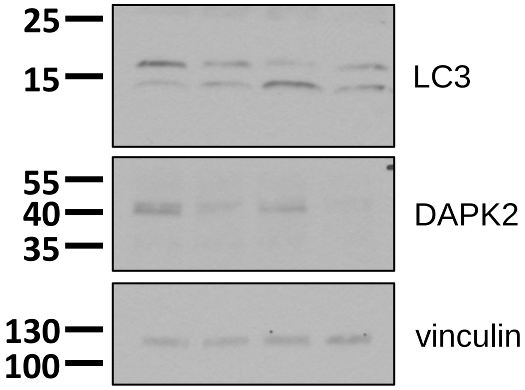

Figure 8b

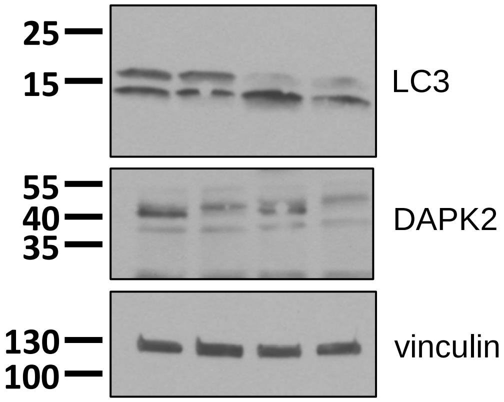

Supplementary Figure 7. Uncropped images of immunoblots**.**

Uncropped images of several important immunoblots displayed in the main figures of the manuscript. Molecular weight markers displayed on the left side. Where more than one clear band is visible, red boxes mark the relevant protein. When running western blots of cell lysates that were reacted with antibodies against proteins with similar molecular weights, one mix was prepared and samples were run in more than one gel.
